# Marine-derived myxobacteria of the suborder Nannocystineae: An underexplored source of structurally intriguing and biologically active metabolites

**DOI:** 10.3762/bjoc.12.96

**Published:** 2016-05-13

**Authors:** Antonio Dávila-Céspedes, Peter Hufendiek, Max Crüsemann, Till F Schäberle, Gabriele M König

**Affiliations:** 1 Institute for Pharmaceutical Biology, University of Bonn, Nussallee 6, 53115 Bonn, Germany

**Keywords:** *Enhygromyxa*, genome mining, myxobacteria, Nannocystineae, natural products

## Abstract

Myxobacteria are famous for their ability to produce most intriguing secondary metabolites. Till recently, only terrestrial myxobacteria were in the focus of research. In this review, however, we discuss marine-derived myxobacteria, which are particularly interesting due to their relatively recent discovery and due to the fact that their very existence was called into question. The to-date-explored members of these halophilic or halotolerant myxobacteria are all grouped into the suborder Nannocystineae. Few of them were chemically investigated revealing around 11 structural types belonging to the polyketide, non-ribosomal peptide, hybrids thereof or terpenoid class of secondary metabolites. A most unusual structural type is represented by salimabromide from *Enhygromyxa salina*. In silico analyses were carried out on the available genome sequences of four bacterial members of the Nannocystineae, revealing the biosynthetic potential of these bacteria.

## Review

### Taxonomy and ecology of myxobacteria

Bacteria from the order Myxococcales, commonly known as myxobacteria, are Gram-negative, rod-shaped δ-proteobacteria, comprising compared to many other bacteria large genomes. The 14,782,125 bp large genome of *Sorangium cellulosum* So0157-2 is the largest bacterial genome reported to date [1]. Myxobacteria are able to glide over surfaces in swarms in order to facilitate heterotrophic nutrition on macromolecules as well as on whole microorganisms, a distinctive trait of these bacteria [2]. Additionally, under adverse environmental conditions, e.g., nutrient shortages, high temperature and dryness, individuals cooperate to create intercommunicated multicellular myxospore-containing fruiting bodies in order to ensure the distribution of nutrients they can still harness, allowing germination as soon as conditions are favorable for the vegetative phase again [3]. Based on our experience in the laboratory, fruiting bodies can occur on solid surfaces like agar plates, as well as in liquid cultures. These adaptive strategies play a fundamental role on how these organisms are able to endure under unfavorable conditions [2,4]. In terms of oxygen demand for growth, myxobacteria were thought to be strictly aerobic until the only anaerobic genus known to date, *Anaeromyxobacter*, was reported in 2002 [5]. Also, for many years myxobacteria were considered to typically occur only in terrestrial habitats and much has been published in terms of their morphology, physiology and ecology [2,4,6–7].

Recently, ever more myxobacteria from intertidal and marine environments were reported. According to salt requirements for growth, a straightforward classification of these bacteria has been provided [8]: (I) halotolerant strains are capable to grow with or without NaCl; and (II) halophilic bacteria are unable to grow without sea salt. Bacteria of group (I) may be derived from terrestrial organisms, which have adapted to saline conditions, whereas those of group (II) may be of truly marine origin.

A typical halotolerant myxobacterium, originally obtained from coastal samples, is the *Myxococcus fulvus* strain HW-1 (suborder Cystobacterineae). In this case, it was demonstrated that salt concentration not only affects growth, but also myxobacterial motility systems as well as fruiting body formation [9]. In the absence of salt, both capabilities were diminished. Mutational studies imply that the single, probably horizontally transferred gene *hdsp*, which was found in five halotolerant *Myxococcus* strains, but not in soil-derived *Myxococcus* strains, leads among other changes to sea water tolerance [10].

Only few myxobacteria dwelling in sea habitats are considered as halophilic, that is, of true marine origin. Indeed, early isolates from marine environments were thought to be halotolerant terrestrial myxobacteria whose myxospores had been washed into the ocean [11]. This opinion prevailed until Fudou and Iizuka [12–15] discovered the first strictly halophilic myxobacteria within the suborder Nannocystineae, namely *Enhygromyxa*, *Haliangium*, and *Plesiocystis*, which strictly require sea-like salinity conditions in order to grow. Whether the adaptation to the marine environment was acquired independently several times, or if these entire marine clades share one common ancestor, is not clarified yet due to the relatively low number of species known to date.

Regarding the strategies employed by these bacteria to cope with salt and desiccation stress, the accumulation of organic osmolytes instead of the salt-in strategy can be expected. This hypothesis is supported by the fact that all strains investigated so far can grow within a relatively wide range of salinity. Also, the terrestrial strain *M. xanthus*, which is slightly halotolerant, uses organic osmolytes, i.e., glycine betaine to combat osmotic stress [16]. Analyses on the osmoprotective strategies of *Enhygromyxa salina* SWB007 and *Plesiocystis pacifica* SIR-1 revealed that both closely related strains rely on organic osmolytes. For instance, *E. salina* SWB007 biosynthesizes the osmolytes betaine, ectoine, and especially hydroxyectoine under high salt concentrations. In contrast, *P. pacifica* SIR-1 does not synthesize specialized compatible solutes; this strain rather accumulates amino acids as osmoprotective agents [17]. Of course, further mechanisms may be involved for osmoregulation, but are not known to date for myxobacteria. A list of general bacterial osmoregulation processes is given elsewhere [18].

### Secondary metabolites from myxobacteria

**Figure 1:**
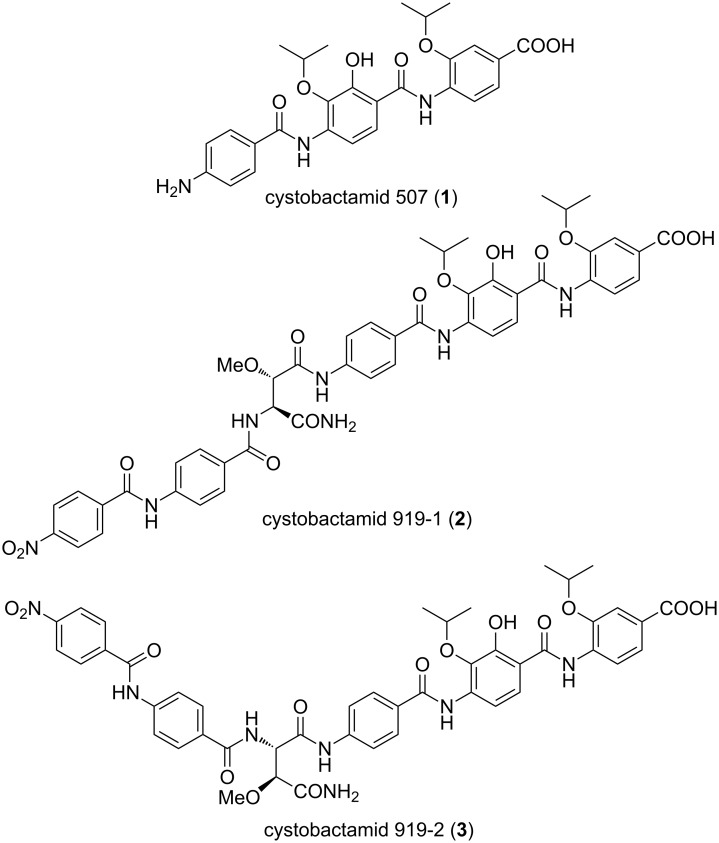
Structures of cystobactamids 507, 919-1 and 919-2.

**Figure 2:**
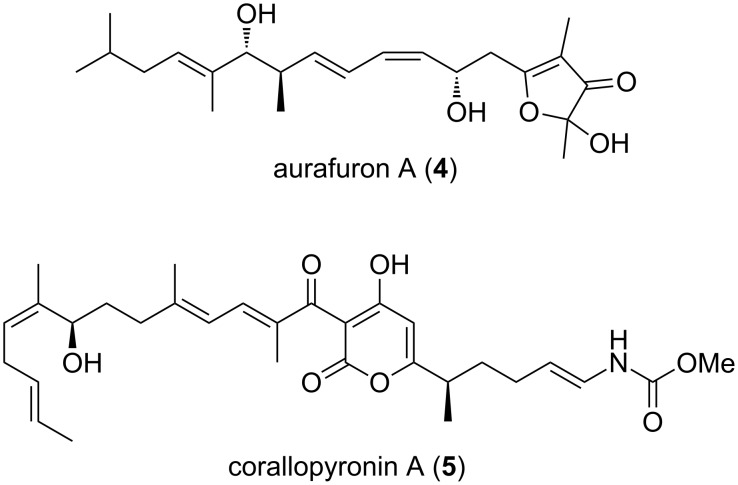
Structures of aurafuron A and corallopyronin A.

Members of the order Myxococcales are famous for their ability to produce secondary metabolites of diverse chemical nature with the capability to exert different biological effects [19–20]. Detailed descriptions of myxobacteria-derived metabolites can be found in various detailed reports [19–23]. The majority of these metabolites are either non-ribosomal peptides, e.g., cystobactamids **1–3** (Figure 1) [24], polyketides, e.g., aurafuron A (**4**) [25], or hybrids thereof, e.g., corallopyronin A (**5**, Figure 2) [26].

**Figure 3:**
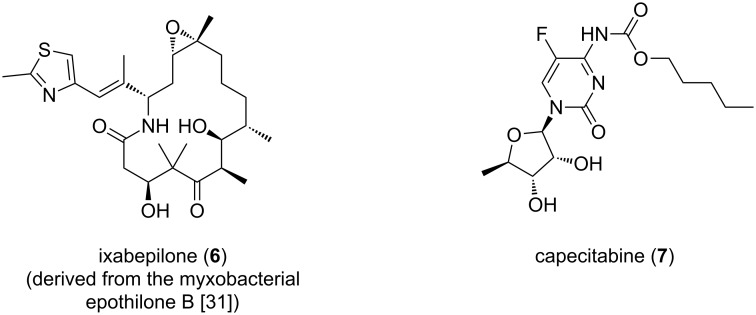
Structures of ixabepilone and capecitabine.

Interestingly, an ample amount of them was shown to work as antimicrobial agents, above all corallopyronin A. This is probably a reflection of their predatory habits [27–28]. A comprehensive description of antibiotics obtained from myxobacteria can be found in a previous review [21]. One outstanding example of a biologically active PKS/NRPS-derived compound produced by the terrestrial *S. cellulosum* is the microtubule stabilizer epothilone B, of which the lactam analogue ixabepilone (**6**) is currently used together with capecitabine (**7**, Figure 3) in cancer therapy to improve the effectiveness of taxane-resistant metastatic breast cancer treatment, demonstrating the therapeutic potential of myxobacterial secondary metabolites [29–31]. This drug has also been assessed as chemotherapeutic agent in pancreatic lymphoma showing promising results and tolerable toxicity [32].

Over time, different strategies have been designed to tackle the sometimes cumbersome task of finding bioactive secondary metabolites of bacterial origin, i.e., mainly bioactivity- or chemistry-guided methods. An additional approach, complementary to the latter, arose in the late 1990s, when the first microbial genomes were sequenced, allowing genome mining. Thus, knowledge derived from bioinformatic analysis of microbial genomes paved the way to gain detailed insights into bacterial secondary metabolism. Since then, in silico methods for genome mining have enormously advanced and effective experimental approaches for connecting genomic and metabolic information have been developed [33–35].

**Figure 4:**
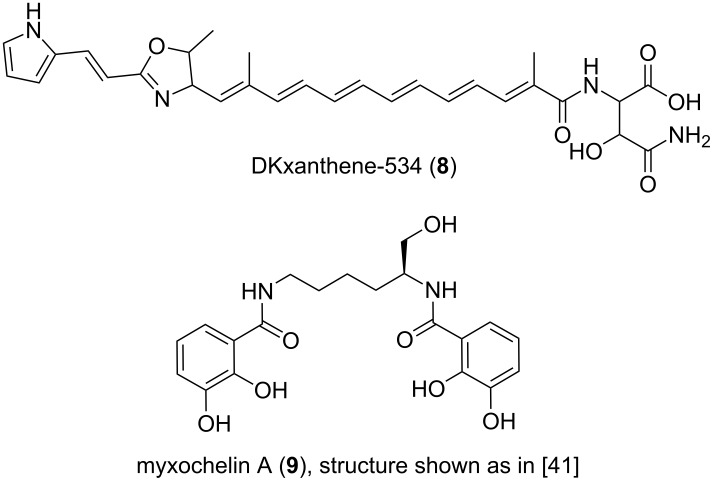
Structures of DKxanthene-534 and myxochelin A.

The terrestrial *Myxococcus xanthus* strain DK1622 was the first myxobacterium to have its 9.14 Mb genome sequenced, with the initial aim to study its swarming motility and fruiting body formation [36]. At the same time, this work reinforced the notion that large genomes often correlate with the potential for prolific secondary metabolite production by revealing almost 8.6% of the genome to be possibly involved in the biosynthesis of secondary metabolites [36]. Mining this genome for the presence of PKS and NRPS genes exposed 18 biosynthetic clusters, with a predominance of hybrid PKS-NRPS systems [37]. *M. xanthus* strain DK1622 is responsible for the synthesis of metabolites like DKxanthene-534 (**8**, Figure 4), a pigment required for fruiting body formation and sporulation processes [37] and the siderophore myxochelin A (**9**), which belongs to a class of compounds that have recently been shown to have antiproliferative effects on leukemic K-562 cells [27].

Halotolerant and halophilic marine myxobacteria are poorly investigated regarding secondary metabolite production. Therefore, the pool of secondary metabolites isolated from organisms of this kind is very low to date, when compared to that of their terrestrial counterparts. This is mainly due to the difficulties that are encountered during isolation and cultivation processes of marine myxobacteria. However, herein we intend to show that these organisms are a great research niche, which offers the opportunity to find novel bioactive compounds with the potential to become drug leads.

Regarding halotolerant myxobacteria, the 9 Mb genome of the *Myxococcus fulvus* strain HW-1 (ATCC BAA-855, suborder Cystobacterineae) was the first-of-its-kind to be sequenced [38]. Genome mining performed in our group revealed that this organism displays, analogous to the previously discussed example *M. xanthus* strain DK1622, a plethora of cryptic gene clusters, e.g., five NRPS, four hybrid PKS-NRPS, one PKS-NRPS-lantipeptide, three lantipeptide and numerous bacteriocin- and terpene-encoding gene loci. A more detailed comparison revealed that both strains share the majority of their gene clusters, among them the aforementioned DKxanthene, the myxochromide [39] and myxoprincomide [40] pathways, besides other, yet uncharacterized loci. It is worth mentioning that no gene clusters involved in synthesis of osmolytes like betaine and ectoine were detected in the halotolerant bacterium.

### Taxonomy, cultivation and secondary metabolite chemistry of marine-derived myxobacteria (Nannocystineae)

**Figure 5:**
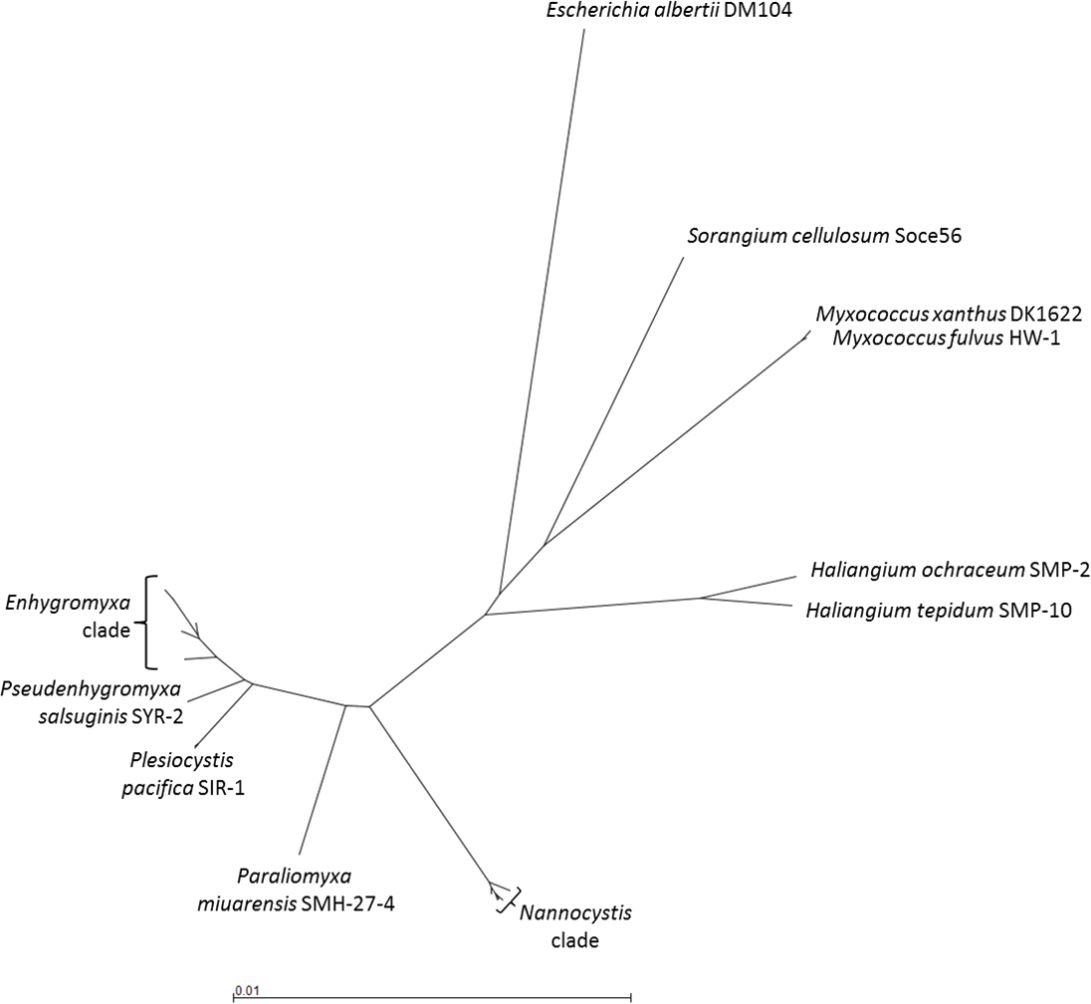
Phylogenetic tree of halotolerant and halophilic myxobacteria. The neighbor-joining tree is based on a multiple sequence alignment (MSA) of the 16S rDNA sequences. The terrestrial myxobacteria *Myxococcus xanthus* DK1622 and *Sorangium cellulosum* Soce56 as well as *Escherichia albertii* DM104 are included for comparison (see Supporting Information File 1 for the sequences used; the MSA was computed using Clustal Omega).

The following sections summarize features of three halotolerant and three marine-derived bacterial taxa clustered in the suborder Nannocystineae: *Nannocystis, Haliangium, Enhygromyxa, Plesiocystis,* Myxobacterium SMH-27-4 (i.e., *Paraliomyxa*) and *Pseudenhygromyxa* (see Figure 5).

Information on strain isolation, culture conditions, phylogeny, genetics and hitherto isolated molecules will be provided, with an emphasis on structural details and biological activity of the metabolites. Additionally, putative gene clusters for secondary metabolite biosynthesis are included in this review. For this reason we were mining the four available genomes from bacteria of the Nannocystineae using the antiSMASH 3 tool [41] (see Table 1). The genome mining results are generated by feeding the software with the INSDC code of the annotated sequences. The databases are continuously updated, thus, results presented in this part of the review may vary in ulterior analyses. Our data, however, present a useful guide for future projects. For detailed information on terrestrial myxobacteria and other predatory bacteria, readers are referred to the review article from Korp et al. in this thematic series [42].

**Table 1:** Summary of antiSMASH analysis (version 3.0.4) of the four available genomes of myxobacteria of the suborder Nannocystineae.

	halophilic/halotolerant			terrestrial
	
	*Enhygromyxa salina* DSM 15201	*Plesiocystis pacifica* SIR 1	*Haliangium ochraceum* DSM 14365	*Nannocystis exedens* ATCC 25963

genome size (Mb)	10.44	10.59	9.45	11.61
GC %	67.4	70.7	69.5	72.2
number of contigs	330	237	1	174
% of genome involved in secondary metabolism^a^	9.2	6.4	10.1	8.2
total number of clusters	38	28	25	31
NRPS	2	1	3	1
PKS (including PKS hybrids)	13	11	2	2
NRPS/PKS hybrids	2	0	3	6
terpene	7	6	3	10
bacteriocin	6	6	5	3
ribosomal peptides	0	0	4	1
siderophore	2	1	0	2
indole	1	0	0	0
arylpolyene	2	1	0	2
phenazine	0	0	0	2
ectoine	0	0	1	0
other	3	2	4	2

^a^Total bases of all detected antiSMASH secondary metabolite gene clusters divided by number of bases in the genome.

#### The genus *Nannocystis*

**Figure 6:**
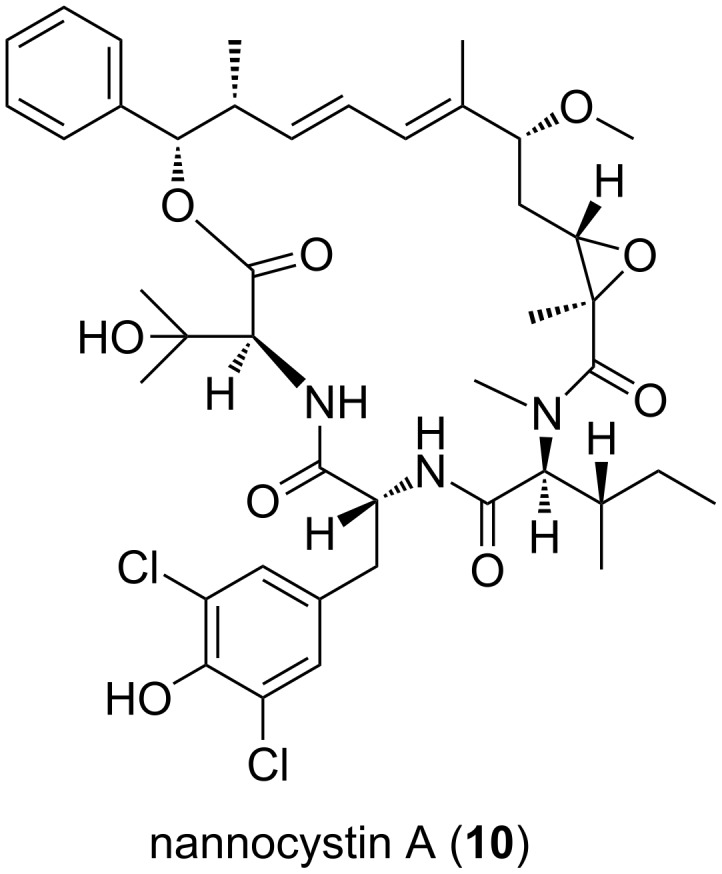
Structure of nannocystin A.

Bacteria of the genus *Nannocystis* are merely halotolerant and frequently isolated from terrestrial or intertidal regions. Back in the 1970s, the Reichenbach group informed on the isolation of a widely distributed soil-dwelling myxobacterium similar to members of the genus *Sorangium* in terms of cytology and growth pattern [43]. After taxonomic studies, they proposed a new genus and species, i.e., *Nannocystis exedens.* Only recently it was recognized that members of this genus have a great potential as producers of metabolites with relevant biological activities. One striking example is the 2015 described nannocystin A (**10**, Figure 6), a macrocyclic compound of NRPS-PKS origin with strong antiproliferative properties isolated from the terrestrial *Nannocystis* sp. ST201196 (DSM 18870) [44].

**Figure 7:**
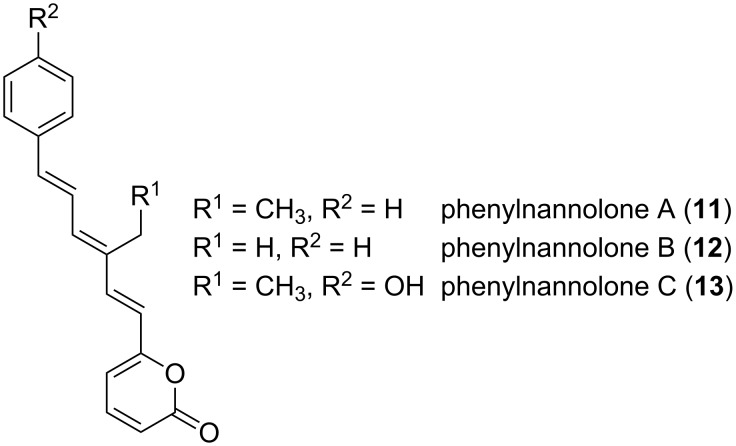
Structure of phenylnannolones A–C.

It was subsequently shown that nannocystin A targets the eukaryotic translation elongation factor 1α, a promising novel target for cancer therapy [44]. Regarding metabolites from halotolerant *Nannocystis* strains, the most outstanding examples are the phenylnannolones A–C (**11–13**, Figure 7), molecules of polyketide nature with a phenylalanine-derived starter unit [45].

These compounds are synthesized by *N. exedens* strain 150, later reassigned to *N. pusilla,* isolated from the intertidal region of a beach in Crete [45]. Cultivation of the regarding organism was carried out in liquid medium with addition of adsorber resin. The adsorbed metabolites were then extracted and isolated via different chromatographic techniques. Phenylnannolone A (**11**) was found to be the main metabolite, which is accompanied by only minute amounts of the other derivatives **12** and **13**. Phenylnannolone A was proven to restore daunorubicin sensitivity in cancer cells by inhibiting P-glycoprotein (P-gp), an ATP-binding cassette transporter (ABC transporter). In tumor cells, P-gp serves as an efflux transporter of drugs, ultimately leading to treatment failure [46].

**Figure 8:**
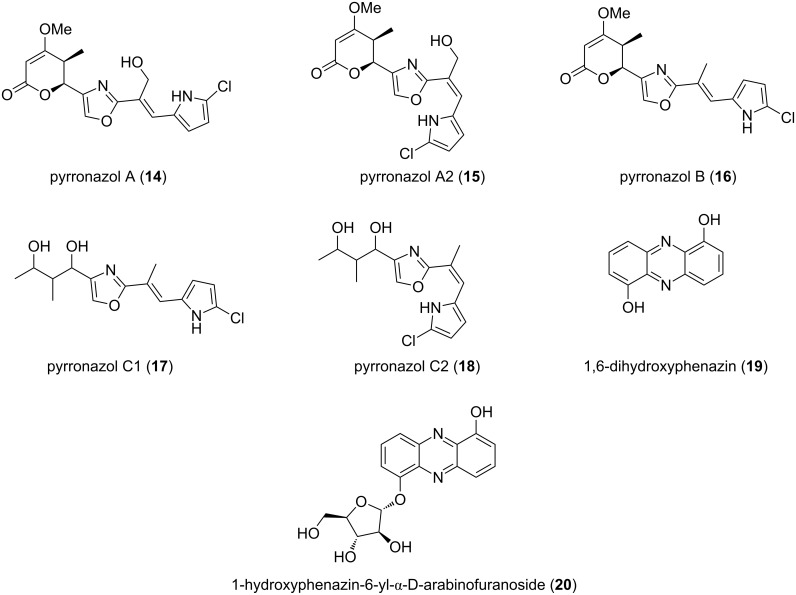
Structures of the pyrronazols, dihydroxyphenazin and 1-hydroxyphenazin-6-yl-α-D-arabinofuranoside.

Apart from these polyketides, an array of nitrogen-containing metabolites was published recently by Jansen et al. [47]. They investigated three strains of *N. pusilla* isolated from coastal sediment samples – deemed halotolerant for this reason – for their secondary metabolite production. Two strains, Ari7 and Na a174 yielded a new class of halogenated pyrrole–oxazole compounds **14–18** (Figure 8). Pyrronazols A (**14**), A2 (**15**) and B (**16**), synthesized by Ari7, additionally include an α-pyrone moiety [48].

This is worth noting, since the non-cyclic alkyl moiety in the pyrronazols C1 (**17**) and C2 (**18**), found in strain Na a174, is supposed to be a result of degradation processes due to long-time cultivation. Additionally, the known compound 1,6-dihydroxyphenazin (**19**) and its arabinofuranoside **20** were obtained.

To date, **14** has shown marginal antifungal activity towards *Mucor hiemalis* (DSM 2656, MIC: 33.3 μg/mL) and **19** proved to have cytotoxic effects in the lower micromolar range against different cancer cell lines [47].

**Figure 9:**
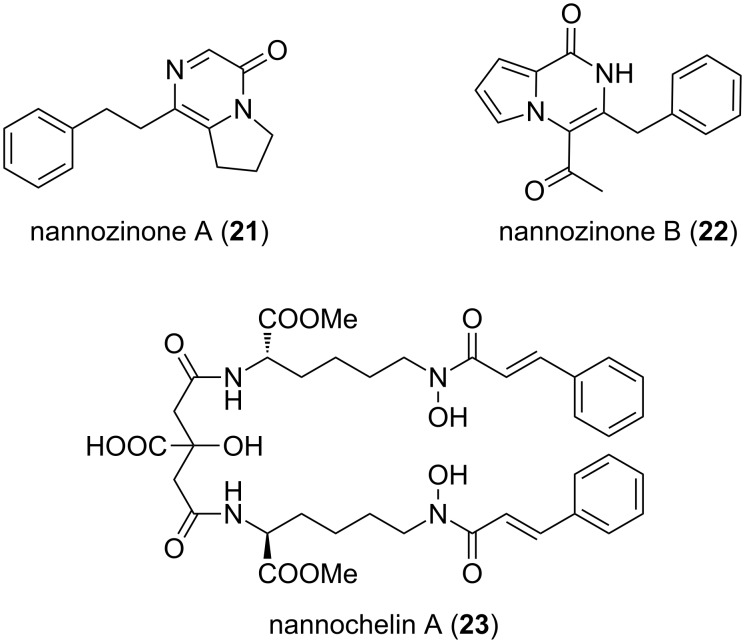
Structures of nannozinones A + B and nannochelin A from *N. pusilla* strain MNa10913.

Nannozinones A (**21**) and B (**22**, Figure 9) are produced by *N. pusilla* strain MNa10913, isolated from a soil sample, collected in Mallorca, Spain [49]. They represent novel pyrazinone type molecules. Additionally, the siderophore nannochelin A (**23**) also from other myxobacteria was isolated [50].

All three compounds were tested against a broad range of microorganisms and mammalian cell lines. The most relevant antimicrobial activity was shown by **21** towards *Mycobacterium diernhoferi* (DSM 43542), *Candida albicans* (DSM 1665) and *Mucor hiemalis* (DSM 2656, 33.3 μg mL^−1^ in each case). Significant cytotoxic activity was revealed for **22** towards SKOV-3 (IC_50_ = 2.4 μM), KB3-1 (IC_50_ = 5.3 μM), as well as A431 (IC_50_ = 8.45 μM), while **23** showed remarkable cytotoxicity against cell lines HUVEC and KB3-1 (IC_50_ = 50 nM).

So far, no genome sequence is publicly available for halotolerant strains of this genus. The only sequence found in the databases belongs to the terrestrial *N. exedens* ATCC 25963 (see Table 1). Remarkably, this genome encodes, among a considerable number of NRPS/PKS hybrids, 10 different terpene biosynthesis gene clusters.

#### The genus *Haliangium*

*Haliangium ochraceum* sp. nov. (initially termed *H. luteum*, DSM 14365^T^) and *H. tepidum* sp. nov. (DSM 14436^T^) were isolated from seaweed and sea grass, respectively, with both samples being obtained from a sandy beach in Miura, Japan by Fudou et al. in 2002 [12]. The species were proposed to be of true marine origin according to their salt requirement for growth. Indeed, 2 to 3% NaCl (w/v) and a pH of 7.5 are optimal for growth on yeast medium with artificial seawater solution. These conditions were established for routine isolation and cultivation. One particular feature worth mentioning is that the species have rather different optimal growth temperature intervals: 30–34 °C for *H. ochraceum* and 37–40 °C for *H. tepidum*. According to the original report, both strains share 95.5% of 16S rDNA sequence identity and have the terrestrial *Kofleria flava* (DSM 14601) as their closest relative (16S rDNA sequence identity lower than 95%). The GC content of *H. ochraceum* and *H. tepidum* is 67 and 69 mol %, respectively.

**Figure 10:**
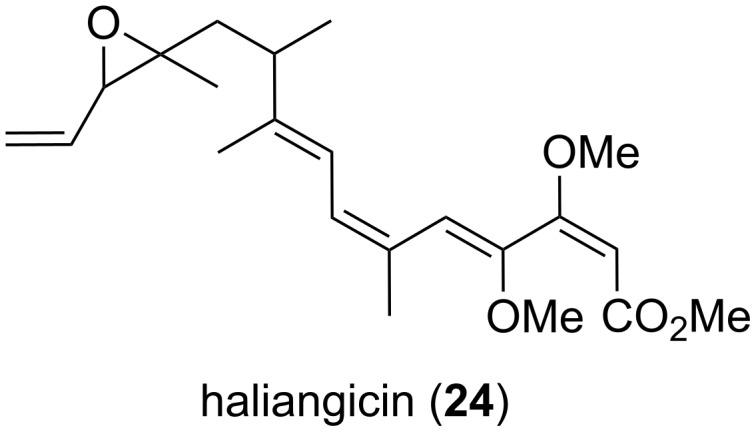
Structure of haliangicin from *H. ochraceum.*

*H. ochraceum* was found by Fudou et al. [51] to be a producer of secondary metabolites with antibacterial and antifungal activities. Further investigation led to the isolation and structure elucidation of the bioactive polyketide haliangicin (**24**, Figure 10), which was the first myxobacterial metabolite of true marine origin.

It was found that this molecule comprised a β-methoxyacrylate subunit including a conjugated tetraene moiety [52]. The complete structure was elucidated via 2D NMR techniques, while NOESY correlations were used to study the configuration of the double bonds. However, the configuration at the epoxide bearing carbon atoms could not be resolved at that time. In further work, Kundim et al. [53] published three new haliangicin stereoisomers, which differed in the configuration of the three terminal double bonds in the tetraene moiety. Each of the isomers haliangicin, haliangicin B, haliangicin C and haliangicin D happened to be present with two different configurations around the epoxy group. The NOESY spectra showed correlations for the *cis*- and *trans*-configuration. However, the absolute configuration of the chiral centers could not be resolved yet. Moreover, the origin of the different isomers is not clear. Due to their alleged instability upon exposure to air and light, one could speculate that isomerization occurs during the purification process. Interestingly, the authors found a NaCl-dependent production of haliangicin [51]. The optimal production range is at 2–3% NaCl (w/v) in the medium, which is the same range as for optimal growth. Haliangicin showed activity against some fungal organisms, e.g., *Aspergillus niger* (AJ117374, MIC: 12.5 μg mL^−1^) and *Fusarium* sp. (AJ177167, MIC: 6.3 μg mL^−1^). These MIC values were in a similar range as those of known antifungal compounds such as amphotericin B or nystatin against the same fungi (MIC: 3.1 μg mL^−1^ for both compounds).

**Figure 11:**
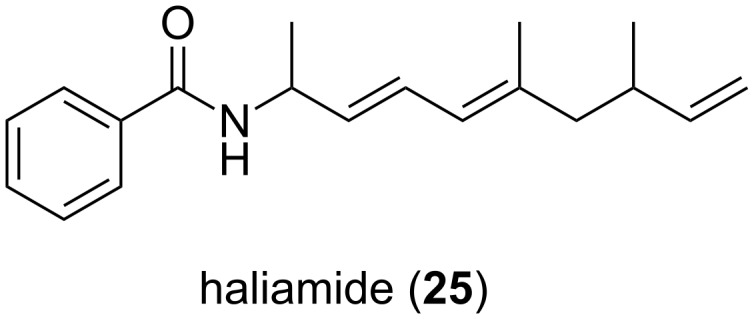
Structure of haliamide from *H. ochraceum* SMP-2.

Recently, the 9.4 Mb genome of *H. ochraceum* (DSM 14365^T^) was completely sequenced and published [54]. This was the first marine-derived myxobacterium to have its genome fully determined. We performed an antiSMASH analysis on the genome of *H. ochraceum* (DSM 14365^T^, INSDC: CP001804.1). The results revealed the presence of 25 secondary metabolite gene clusters, among them three NRPS, two PKS, three NRPS/PKS and four ribosomal peptides (see Table 1). Apart from homologies to geosmin (100%), aurafuron (71%) and paneibactin (50%) biosynthetic genes, the gene clusters display a large degree of novelty. Recently, the biosynthetic gene cluster of haliangicin was heterologously expressed in *Myxococcus xanthus*, leading to tenfold higher haliangicin production than in the native producer. Insights into its biosynthesis were gained by feeding studies with labeled precursors and in vitro experiments. Additionally, unnatural haliangicin analogues that provided insights into the structure–activity relationship of haliangicin were generated in this study [55]. The huge potential to synthesize novel metabolites in the genus *Haliangium* is further corroborated by a PCR screening-based study for PKS sequences in *H. tepidum* among other myxobacteria [56]. The authors found *H. tepidum* to contain the highest amount of novel PKS sequences in this array. Additionally, the indications at the genetic level are reinforced by the very recent discovery of a new compound produced by *H. ochraceum* SMP-2, i.e., haliamide (**25**, Figure 11). The authors also describe the corresponding hybrid PKS-NRPS machinery responsible for metabolite biosynthesis [57]. The molecule was shown to have cytotoxic effects towards HeLa-S3 cells (IC_50_ = 12 μM).

#### The genus *Enhygromyxa*

All *Enhygromyxa* species isolated to date are halophilic and considered as truly marine myxobacteria. The initial report on isolation, characterization and taxonomic classification of six strains of *E. salina,* SHK-1^T^, SMK-1-1, SMK-1-3, SMK-10, SKK-2, and SMP-6, was published in 2003 by Iizuka et al. [14]. These organisms were obtained respectively from mud, sand and algal samples collected in marine environments around Japan. Later, four additional strains of *E. salina* were isolated by the group of König from marine-intertidal sediment samples collected at the West Coast of the USA, at German coasts and the Netherlands [58–59]. As judged from these varied geographical occurrences a world-wide distribution of *Enhygromyxa* species is likely.

The prior mentioned groups reported 1–2% NaCl (w/v) and a pH interval of 7.0–8.5 for optimal growth on yeast medium. The optimal temperature for growth was determined as 28–30 °C. In terms of gliding motility, fruiting body and myxospore formation, these bacteria show the typical features of terrestrial myxobacteria. A salt-dependency and high G + C content ranging from 65.6 to 67.4 mol % [14] and 63.0 to 67.3 mol % [59] were also observed. Based on 16S rDNA sequence alignments, *E. salina* SHK-1^T^ (NR_024807) from Iizuka [14] was found to be most closely related to the *E. salina* strains from the König group [59]. The 16SrDNA sequences of these strains share between 98% (SWB004, AN: HM769727) and 99% (SWB005, AN: HM769728; SWB006, AN: HM769729; SWB007, AN: KC818422) identity.

Some of the bacterial isolates were originally evaluated for their ability to produce PKS-type metabolites and for the biosynthesis of antimicrobials [59]. In these studies, PKS genes could be amplified, sequenced and compared with known sequences in the BLAST database. The potential of producing active molecules was established by using disc diffusion antibiotic activity testing of the bacterial extracts, whereby an inhibitory effect of the extracts of *E. salina* SWB005 on various test organisms, including the clinically relevant MRSA (methicillin resistant *Staphylococcus aureus*) strains LT1334, LT1338 and MRSE methicillin resistant *Staphylococcus epidermidis strain* LT1324 was observed [59].

**Figure 12:**
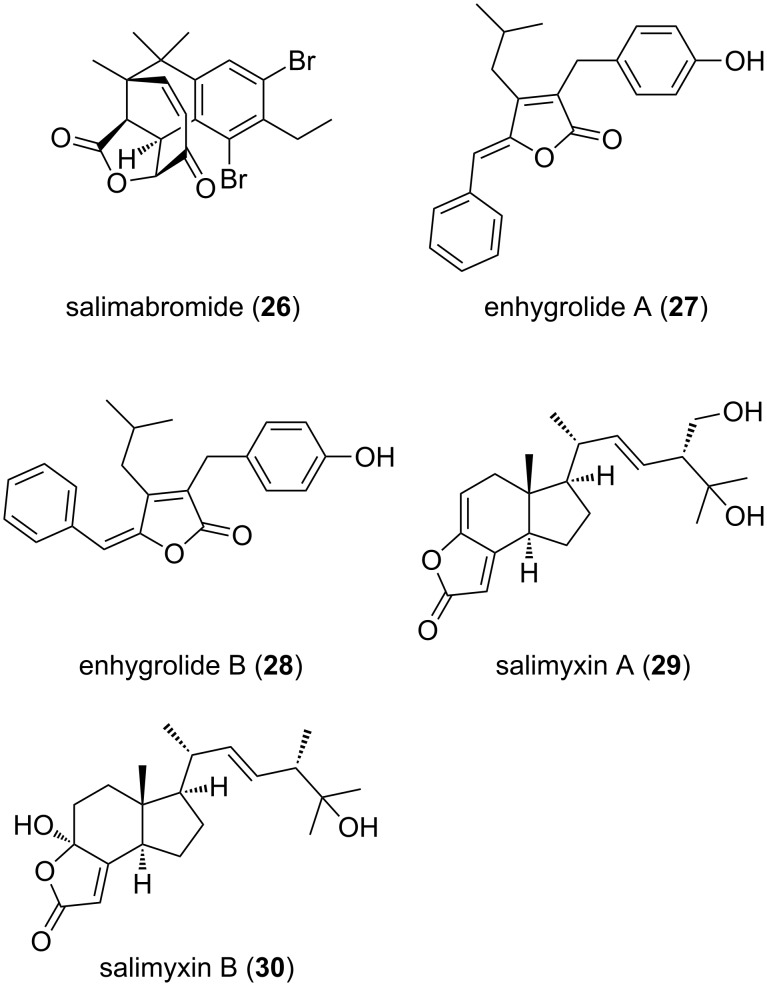
Structures of salimabromide, enhygrolides A + B and salimyxins A + B.

To date, five structures of putative polyketide, shikimate and terpenoid origin have been described and classified as salimabromide (**26**, Figure 12) [58], produced by *E. salina* SWB007, enhygrolides **27** and **28** and salimyxins **29** and **30** produced by *E. salina* SWB005 [60].

Salimabromide (**26**) is particularly interesting due to its unique halogenated tetracyclic core structure. Its biosynthesis is postulated to be carried out by a type III-PKS. Pure PKS-derived compounds are rare in myxobacteria, which together with the new carbon skeleton and the high bromination level makes this structure even more fascinating. Structure elucidation was achieved via extensive NMR measurements. The absolute configuration of the chiral centers could be resolved through comparison of the experimental CD spectrum with calculated data. This metabolite showed inhibitory activity towards the bacterium *Arthrobacter crystallopoietes* with an MIC value of 16 μg mL^−1^. Further bioactivity assays were impossible to carry out due to the minute amounts in which this metabolite is produced. Consequently, a synthetic approach has been utilized in order to overcome this problem, leading so far to the synthesis of the tricyclic core structure of the molecule. However, the complete natural product has not yet been synthesized [61].

The enhygrolide group of compounds comprises enhygrolide A (**27**) and B (**28**). These molecules resemble cyanobacteria-derived metabolites, known as nostoclides and cyanobacterin, which also have a γ-lactone-moiety with a similar substitution pattern. In the myxobacterial metabolites an *E*-configuration was found at the benzylidene unit, whereas the nostoclides and cyanobacterin have a *Z*-configuration. Only the anhydro form of cyanobacterin was found to isomerize from the *Z*- to the *E*-configured isomer in organic solvents upon light exposure [62].

The salimyxins represent the third group of compounds, i.e., salimyxin A (**29**) and B (**30**). These belong to a subgroup of terpenoids named incisterols, which were first discovered from the sponge *Dictyonella incisa* [63]. Their biosynthesis presumably involves oxidative degradation of a sterol, leading to the tricyclic core structure. Compounds **27** and **30** have shown inhibitory activity towards *A. crystallopoietes* (MIC value of 8 and 4 μg mL^−1^, respectively).

Since salimabromide is a novel structure of putatively assigned PKS origin, our research group sequenced the genome of the producing strain SWB007. The results revealed a PKS III gene cluster adjacent to a halogenase sequence (unpublished data). Upon sequencing, primers for these genes were designed. Given the genetic proximity among the *E. salina* isolates, strains SWB004, SWB005 and SWB006 were screened with the PKS III and halogenase primers specific for the SWB007 sequence revealing the presence of close-to-identical sequences in their genomes (unpublished data), a clear suggestion that similar molecules may be also produced by the related strains.

Bioinformatic analysis was performed by us on the available genome of *E. salina* (DSM 15201, INSDC: JMCC00000000.2) of which as to date, no reports on the isolation of compounds are available. However, the screening with antiSMASH revealed an ample amount of uncharacterized biosynthetic gene clusters, among them 17 gene clusters putatively responsible for the synthesis of NRPS, PKS and hybrid NRPS-PKS products (see Table 1). Apart from the geosmin biosynthesis genes, no gene cluster or fragment thereof shares an identity higher than 33% to any known gene cluster in the MiBIG database [64]. It should be noted that since this is a draft genome sequence, the actual amount of gene clusters might be slightly smaller, since small contigs display only fragments of gene clusters.

#### The genus *Plesiocystis*

In 2003 Iizuka et al. [13] proposed the genus and the species *Plesiocystis pacifica* for the myxobacterial strains SHI-1 (JCM 11592, DSM 14876) and SIR-1^T^ (JCM 11591^T^, DSM 14875T). SHI-1 was retrieved from a sand sample of a Japanese coastal area, whilst strain SIR-1^T^ was isolated from a piece of dried marine grass (*Zostera* sp.). These strains require 2–3% NaCl (w/v) and a pH of 7.4 for optimal growth on yeast medium with artificial seawater solution at 28 °C.

According to Iizuka et al. [13] the isolates are closely related, sharing 99.5% 16S rDNA sequence identity between them. Their closest relative was reported to be *Nannocystis exedens* DSM 71T, with 89.3% sequence identity to SIR-1T and 89.4% to SHI-1. Regarding GC content, SIR-1T and SHI-1 were reported to have 69.3 and 70.0 mol %, respectively. Such a high GC content is a distinctive trait of all myxobacteria. To date, no reports on biological testing of extracts or of any metabolites isolated from these organisms have been published.

The antiSMASH analysis was performed on the available draft genome of *P. pacifica* strain SIR-1 (INSDC: ABCS00000000.1). The analysis revealed the presence of 12 NRPS, PKS and hybrid NRPS-PKS gene clusters amongst many others (see Table 1), which should encourage researchers to isolate some of the predicted metabolites. Here, no gene clusters sharing more than 28% identity to pathways of characterized molecules were detected. Again, many of the PKS and NRPS gene cluster appear to be fragmented to smaller contigs.

#### Myxobacterium SMH-27-4 (*Paraliomyxa miuraensis*)

In 2006, as a result of the remarkable efforts of Iizuka et al. [65] a novel myxobacterium was isolated from a soil sample of a seashore area in Miura, Japan. After genetic analysis, the new isolate was classified as strain SMH-27-4, tentatively named *Paraliomyxa miuraensis*. This strain is considered slightly halophilic.

Yeast medium with only a low sea salt concentration was selected for isolation, whereas cultivation in NaBr-containing medium was selected for antibiotic production. The diminished strength of salinity in the medium implies an optimal salt range concentration for growth of 0.5–1% (w/v), at pH 7.2 and a temperature of 27 °C. Fermentation and the production of antibiotic compounds were performed at 27 °C at pH 7.3.

Surprisingly, the authors do not report fruiting body formation during the cultivation of myxobacterium SMH-27-4, which usually is a hallmark feature of myxobacteria. Phylogenetic analyses carried out by Iizuka et al. [65] revealed that this organism shares 93.0% identity with *Nannocystis exedens* DSM 71^T^ (AB084253), 93.2 to 93.3% with *Enhygromyxa salina* JCM 11769^T^ (AB097590), and 91.3 to 91.5% with *Plesiocystis pacifica* JCM 11591^T^ (AB083432). No information on the GC content of the bacterial genome has been found.

**Figure 13:**
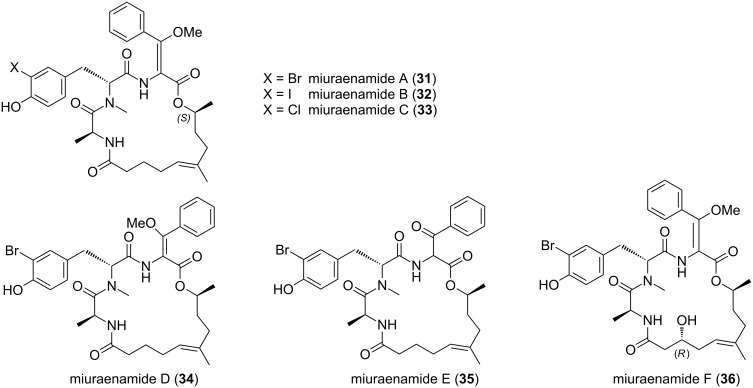
Structures of miuraenamides A–F from *P. miuraensis*.

Myxobacterium SMH-27-4 (AN: AB252740) was investigated for the production of secondary metabolites [65]. This work led to the isolation and structure elucidation of two compounds of peptidic nature called miuraenamide A (**31**) and B (**32**, Figure 13). Two years later, the same research group published the structures of four additional derivatives, named miuraenamides C–F (**33–36**) [66].

The cyclic core structure of this compound class represents a halogenated depsipeptide with an additional polyketide-derived moiety. As in haliangicin, all miuraenamides, except E, contain a methoxyacrylate structural motif, which surely is important for the biological activity. For miuraenamide A (**31**) the absolute configuration was determined. Marfey´s method was employed to show that L-alanine and *N*-methyl-D-tyrosine are present. Furthermore, after acid hydrolysis, methylation and application of modified Mosher´s method the absolute configuration at the oxygen bearing C-9 was deduced as *S*. For miuraenamide F (**36**) the configuration at C-3 was found to be *R*, again using Mosher’s method. For all the other chiral centers in compounds **32–36** the authors conclude that they have the same configuration as determined for **31**. Further work led to semi-synthetic derivatives, which upon activity testing, revealed that the lactone moiety and the configuration of the methoxyacrylate partial structure are crucial for antifungal activity. The latter structural motif is well known from a range of antifungal compounds produced by fungi and myxobacteria, e.g., the strobilurins and the melithiazols [67–68].

Miuraenamide A (**31**) was assayed against various fungal, yeast and bacterial organisms. It showed a remarkable inhibitory effect toward the fungal phytopathogen *Phytophthora capsici* NBRC 8386 (MIC: 0.4 mg mL^−1^) and *Candida rugosa* AJ 14513 (MIC: 12.5 mg mL^−1^). No inhibitory effect on selected bacteria was detected. The mode of action is proposed to be similar to other antifungals with a methoxyacrylate partial structure, i.e., inhibition of the mitochondrial cytochrome bc1 complex. Additionally, miuraenamide A was shown to act as actin filament stabilizer in HeLa cells [69]. The activity of miuraenamide B (**32**) was not assessed due to the minute amounts of the metabolite available.

To date, there is no genome sequence available for this organism.

#### The genus *Pseudenhygromyxa*

*Pseudenhygromyxa salsuginis* is the latest example of a halotolerant myxobacterium, published in 2013 by Iizuka et al. [70]. This organism was retrieved from mud samples of an estuarine marsh in a coastal area in Japan and termed SYR-2^T^. Even though it is able to grow in the absence of salt, optimal growth was shown to occur within a concentration range of 0.2–1.0% NaCl (w/v) and pH values from 7.0–7.5 on CY-S agar (bacto casitone, bacto yeast extract) in a temperature range of 30–35 °C. After alignment of 16S rDNA sequences, Iizuka et al. [70] found that *P. salsuginis* SYR-2^T^ showed 96.5% and 96.0% identity to *Enhygromyxa salina* SHK-1^T^ (NR_024807) and *Plesiocystis pacifica* SIR-1^T^ (NR_024795), respectively. The G + C content is 69.7 mol %, and thus slightly higher than for various *E. salina* strains described above.

To date, no reports on biological activities or any metabolites derived from *P. salsuginis* have been published. Nevertheless, the genetic proximity to *E. salina* and the fact that it belongs to the group of myxobacteria suggests that this organism may also possess a high potential as a producer of active molecules. To date no genome sequence has been published.

## Conclusion

Geographically, halophilic and halotolerant myxobacteria are widely distributed as evidenced by some of the above described bacterial isolates, originating, e.g., from German, Japanese and US American marine environments. This observation is supported by the investigation of myxobacteria-enriched libraries of 16S rRNA gene sequences revealing myxobacteria-related sequences in mud samples taken around Japan. The latter sequences were phylogeographically clearly distinct from those of terrestrial myxobacteria [71]. A further report also states that myxobacteria capable of thriving in ocean-like conditions exhibit a worldwide distribution [72].

The quantitative presence of myxobacteria in marine environments can hardly be judged. Based on the isolation success one may suggest that the frequency is much lower (e.g., only 6 isolates from 90 coastal samples [12,14]) than in terrestrial habitats, but this may simply represent the less than perfect isolation and cultivation conditions used today. Indeed, the currently applied isolation protocols for marine myxobacteria are only slightly altered in terms of the addition of sea salt, when compared to those for terrestrial strains, e.g., terrestrial *Escherichia coli* is still used as prey. In addition, it surely is difficult to recognize marine myxobacterial colonies after isolation, since their morphological features may deviate from the ones of terrestrial strains and are not well known. Thus, in order to use marine-derived myxobacteria as a source of bioactive metabolites, in-depth studies of their morphology and physiology are necessary.

From the available genetic and chemical data, the potential of halotolerant and halophilic myxobacteria as producers of chemically diverse secondary metabolites is out of question. Salimabromide (**26**) from *E. salina* is an outstanding example of such a molecule having a most unusual new carbon skeleton [58]. The number of molecules obtained so far however is very low (Table 2), especially when compared with the expected metabolites envisioned after bioinformatic analysis of the four available genomes of myxobacteria in the suborder Nannocystineae. All strains harbor at least 25 biosynthetic gene clusters (Table 1), most of them bearing no or very little homology to known biosynthetic genes. Compared to *Myxococcus xanthus* DK1622, the so far sequenced strains dedicate a similar or even larger portion of their genome of up to 10% to secondary metabolism.

**Table 2:** Metabolites reported to date from myxobacteria grouped into the suborder Nannocystineae and their bioactivities.

genus	classification according to salt requirements for growth	metabolites	metabolite [No] bioactivity

*Nannocystis*	terrestrial	nannocystin A (**10**)	**10** antiproliferative activity, MDA-MB231 and its related drug resistant MDA-A1 (IC_50_ = 6.5 and 12 nM respectively), HCT116 (IC_50_ = 1.2 nM) and PC3 (IC_50_ = 1.0 nM)

	halotolerant	phenylnannolone A, B, C (**11**–**13**), pyrronazol A, A2, B, C1, C2 (**14**–**18**), nannozinone A, B (**21**, **22**), nannochelin A (**23**)	**11** reversing drug-resistance of tumor cells **21** *Mycobacterium diernhoferi*, *Candida albicans*, *Mucor hiemalis*, MICs: 33.3 μg mL^−1^ in each case **22** cytotoxicity, SKOV-3 IC_50_ = 2.4 μM, KB3-1 IC_50_ = 5.3 μM and A431 IC_50_ = 8.45 μM **23** cytotoxicity, HUVEC and KB3-1 IC_50_ = 50 nM

*Haliangium*	moderately halophilic	haliangicin (**24**) haliamide (**25**)	**24** *Aspergillus niger*, MIC: 12.5 μg mL^−1^, *Phytophthora capsici,* MIC: 0.4 μg mL^−1^; **25** Cytotoxicity, HeLa-S3, IC_50_ 12 μM

*Enhygromyxa*	halophilic	salimabromide (**26**) enhygrolide A, B (**27**, **28**) salimyxin A, B (**29**, **30**)	**26**, **27**, **30** *Arthrobacter crystallopoietes*, MICs: 16, 8 and 4 μg mL^−1^, respectively

Myxobacterium SMH-27-4 (*Paraliomyxa*)	slightly halophilic	miuraenamide A–F (**31**–**36**)	**31** *Trichophyton mentagrophytes*, MIC: 12.5 μg mL^−1^

*Plesiocystis*	halophilic	no metabolites described	–

*Pseudenhygro-myxa*	halotolerant	no metabolites described	–

These “cryptic” or “silent” gene clusters may be addressed with different strategies [73], e.g., the OSMAC (one strain, many compounds) approach [74] or co-culturing and elicitation techniques [75–77]. Mass spectrometric analysis [78–81], dereplication procedures [35,79], bioinformatic analysis and genetic experiments allow a direct connection of metabolites and gene clusters [33–34,80]. On the other hand, recent advances in the direct cloning of gene clusters and their heterologous expression [82–85] may enable researchers to select their “favorite” gene cluster and express and manipulate it in heterologous hosts for rational secondary metabolite discovery. These techniques offer the chance for an effective metabolomics-guided natural product and genome mining platform.

In conclusion, the application of these promising state-of-the-art approaches to marine myxobacteria should expand our knowledge on novel secondary metabolites in these organisms, and help to systematically unveil the chemistry encoded in their genomes.

## Supporting Information

File 1 List of 16S rDNA sequences used for Figure 5.
