# Supplementary material for: Marine-derived myxobacteria of the suborder Nannocystineae: An underexplored source of structurally intriguing and biologically active metabolites
Source: Beilstein J Org Chem. 2016 May 13;12:969–84. doi: 10.3762/bjoc.12.96 (PMC4902002; doi:10.3762/bjoc.12.96)
Supplement: File 1 — List of 16S rDNA sequences used for Figure 5. [file Beilstein_J_Org_Chem-12-969-s001.pdf]

Supporting Information

for

Marine-derived myxobacteria of the suborder Nannocystineae: An underexplored source of structurally intriguing and biologically active metabolites

Antonio Dávila-Céspedes[¶], Peter Hufendiek[¶], Max Crüsemann[¶], Till F. Schäberle and Gabriele M. König[*]

Address: Institute for Pharmaceutical Biology, University of Bonn, Nussallee 6, 53115 Bonn, Germany

[¶]These authors contributed equally to this work

*Corresponding author

Email: Gabriele M. König - g.koenig@uni-bonn.de

List of 16S rDNA sequences used for Figure 5

>*Nannocystis pusilla* Na p29

1 tgagtttgat cctggctcag gacgaacgtt tgcggcgggc ctaacacatg caagtcgaac

61 gggctagcaa tagtcagtgg cgcacgggtg cgtaacacgt aggtaatcaa ccccttggtt

121 cgggataacg ttctgaaaag gagcgctaat accggacgcg tcttcgggag cttcggctcc

181 tgtcgagaaa gacccgcaag ggttgccgag ggacgagcct gcggcccatc agctagttgg

241 cgaggtaata gctcaccaag gcgaagacgg gtagctggtc tgagaggatg atcagtcaca

301 ctggaactga gacacggtcc agactcctac gggaggcagc agtggggaat attgcgcaat

361 gggcgaaagc ctgacgcagc cacgccgcgt gagcgatgaa ggccttcggg tcgtaaagct

421 ctgtggggag agacgaagaa agcctgtgaa gagcaggcct tgacggtatc tccttagcaa

481 gcaccggcta actccgtgcc agcagccgcg gtaatacgga gggtgcgaac gttgctcgga

541 atcattgggc gtaaagcgca cgtaggcggc ggcgtaagcg ggatgtgaaa gcccagggct

601 caaccctgga agtgcatccc gaactgtgtc gcttgaatct cggaggggga cagagaattc

661 ccggtgtaga ggtgaaattc gtagatatcg ggaggaatac cagtggcgaa ggcgctgtcc

721 tggacgaaga ttgacgctga ggtgcgaaag cgtggggagc aaacaggatt agataccctg

781 ggtagtccac gctgtaaacg atgagtgctg gacggtggag gatttgaccc cttcgctgtc

841 gaagctaacg cgttaagcac tccgcctggg gagtacggtc gcaagactaa aactcaaagg

901 aattgacggg ggcccgcaca agcggtggag catgtggttt aattcgacgc aacgcgcaga

961 accttacctg ggttaaatcc actggaacct ggctgaaagg ctggggtgcc cttcggggag

1021 ccggtgagaa ggtgctgcat ggctgtcgtc agctcgtgtc gtgagatgtt gggttaagtc

1081 ccgcaacgag cgcaacccct atcgccagtt gccaccattg agttgggaac tctggcgaga

1141 ctgccggtct aaaccggagg aaggtgggga cgacgtcaag tcctcatggc ccttatgccc

1201 agggctacac acgtgctaca atggctggta caaagagccg caagcccgcg agggtgagca

1261 aatctcaaaa aaccagtctc agttcggatt gcagtctgca actcgactgc atgaagctgg

1321 aatcgctagt aatcggagat cagcacgctc cggtgaatac gttcccgggc cttgtacaca

1381 ccgcccgtca caccatggga gtcggctgct ccagaagtag gaacctcaac cgcaaggtaa

1441 ggccctacca aggagcggtc ggtgactggg gtgaagtcgt aacaaggtag ccgtagggga

1501 acctgcggct ggatcacctc ctta

>*Nannocystis exedens* Na e571

1 tttgatcctg gctcagagcg aacgtttgcg gcgggcctaa cacatgcaag tcgaacgggc

61 tagcaatagt cagtggcgca cgggtgcgta acacgtaggt aatcaacccc ttggttcggg

121 ataacgttct gaaaggagcg ctaataccgg acgcgtcttc gggagcttcg gctcttgtcg

181 agaaagaccc gcaagggttg ccgagggacg agcctgcggc ccatcagcta gttggcgagg

241 taatagctca ccaaggcgaa gacgggtagc tggtctgaga ggatgatcag tcacactgga

301 actgagacac ggtccagact cctacgggag gcagcagtgg ggaatattgc gcaatgggcg

361 aaagcctgac gcagccacgc cgcgtgagcg atgaaggcct tcgggtcgta aagctctgtg

421 gggagagacg aagaaagcct gtgaagagca ggccttgacg gtatctcctt agcaagcacc

481 ggctaactcc gtgccagcag ccgcggtaat acggagggtg cgaacgttgc tcggaatcat

541 tgggcgtaaa gcgcacgtag gcggcggcgt aagcgggatg tgaaagccca gggctcaacc

601 ctggaagtgc atcccgaact gtgtcgcttg aatctcggag ggggacagag aattcccggt

661 gtagaggtga aattcgtaga tatcgggagg aataccagtg gcgaaggcgc tgtcctggac

721 gaagattgac gctgaggtgc gaaagcgtgg ggagcaaaca ggattagata ccctggtagt

781 ccacgctgta aacgatgagt gctggacggt ggaggatttg accccttcgc tgtcgaagct

841 aacgcgttaa gcactccgcc tggggagtac ggtcgcaaga ctaaaactca aaggaattga

901 cgggggcccg cacaagcggt ggagcatgtg gtttaattcg acgcaacgcg cagaacctta

961 cctgggttaa atccactgga acctggctga aaggctgggg tgcccttcgg ggagccggtg

1021 agaaggtgct gcatggctgt cgtcagctcg tgtcgtgaga tgttgggtta agtcccgcaa

1081 cgagcgcaac ccctatcgcc agttgccacc attgagttgg gaactctggc gagactgccg

1141 gtctaaaacc ggaggaaggt ggggacgacg tcaagtcctc atggccctta tgcccagggc

1201 tacacacgtg ctacaatggc tggtacaaag agccgcaagc ccgcgagggt gagcaaatct

1261 caaaaaacca gtctcagttc ggattgcagt ctgcaactcg actgcatgaa gctggaatcg

1321 ctagtaatcg gagatcagca cgctccggtg aatacgttcc cgggccttgt acacaccgcc

1381 cgtcacacca tgggagtcgg ctgctccaga agtaggaacc tcaaccgcaa ggaaaggccc

1441 taccaaggag cggtcggtga ctggggtgaa gtcgtaacaa ggttgccgta ggggaacctg

1501 cggctggatc acctc

>*Nannocystis pusilla* 14622T

1 gtttgatcct ggctcagagc gaacgtttgc ggcgggccta acacatgcaa gtcgaacggg

61 ctagcaatag tcagtggcgc acgggtgcgt aacacgtagg taatcaaccc cttggttcgg

121 gataacgttc tgaaaggagc gctaataccg gacgcgtctt cgggagcttc ggctcctgtc

181 gagaaagacc cgcaagggtt gccgagggac gagcctgcgg cccatcagct agttggcgag

241 gtaatagctc accaaggcga agacgggtag ctggtctgag aggatgatca gtcacactgg

301 aactgagaca cggtccagac tcctacggga ggcagcagtg gggaatattg cgcaatgggc

361 gaaagcctga cgcagccacg ccgcgtgagc gatgaaggcc ttcgggtcgt aaagctctgt

421 ggggagagac gaagaaagcc tgtgaagagc aggccttgac ggtatctcct tagcaagcac

481 cggctaactc cgtgccagca gccgcggtaa tacggagggt gcgaacgttg ctcggaatca

541 ttgggcgtaa agcgcacgta ggcggcggcg taagcgggat gtgaaagccc agggctcaac

601 cctggaagtg catcccgaac tgtgtcgctt gaatctcgga gggggacaga gaattcccgg

661 tgtagaggtg aaattcgtag atatcgggag gaataccagt ggcgaaggcg ctgtcctgga

721 cgaagattga cgctgaggtg cgaaagcgtg gggagcaaac aggattagat accctggtag

781 tccacgctgt aaacgatgag tgctggacgg tggaggattt gacccctteg ctgtcgaagc

841 taacgcgtta agcactccgc ctggggagta cggtcgcaag actaaaactc aaaggaattg

901 acgggggccc gcacaagcgg tggagcatgt ggtttaattc gacgcaacgc gcagaacctt

961 acctgggtta aatccactgg aacctggctg aaaggctggg gtgcccttcg gggagccggt

1021 gagaaggtgc tgcatggctg tcgtcagctc gtgtcgtgag atgttgggtt aagtcccgca

1081 acgagcgcaa cccctatcgc cagttgccac cattgagttg ggaactctgg cgagactgcc

1141 ggtctaaacc ggaggaaggt ggggacgacg tcaagtcctc atggccctta tgcccagggc

1201 tacacacgtg ctacaatggc tggtacaaag agccgcaagc ccgcgagggt gagcaaatct

1261 caaaaaacca gtctcagttc ggattgcagt ctgcaactcg actgcatgaa gctggaatcg

1321 ctagtaatcg gagatcagca cgctccggtg aatacgttcc ggggccttgt acacaccgcc

1381 cgtcacacca tgggagtcgg ctgctccaga agtaggaacc tcaaccgcaa ggaaaggccc

1441 taccaaggag cggtcggtga ctggggtgaa gtcgtaacaa ggtagccgta ggggaacctg

1501 cggctggatc acctcctt

>*Nannocystis aggregans* Na a1

1 tttgatcctg gctcagagcg aacgtttgcg gcgggcctaa cacatgcaag tcgaacgggc

61 tagcaatagt cagtggcgca cgggtgcgta acacgtaggt aatcaacccc tcggttcggg

121 ataacgttct gaaaggagcg ctaataccgg acgtgtcttc gagagcttcg gctcttgtcg

181 agaaagaccc tcaagggttg ccgagggacg agcctgcggc ccatcagcta gttggcgagg

241 taaaagctca ccaaggcgaa gacgggtagc tggtctgaga ggatgatcag tcacactgga

301 actgagacac ggtccagact cctacgggag gcagcagtgg ggaatattgc gcaatgggcg

361 aaagcctgac gcagccacgc cgcgtgagcg atgaaggcct tcgggtcgta aagctctgtg

421 gggagagacg aagaaagcct gtgaagagca ggccttgacg gtatctcctt agcaagcacc

481 ggctaactcc gtgccagcag ccgcggtaat acggagggtg cgaacgttgc tcggaatcat

541 tgggcgtaaa gcgcacgtag gcggcggcgt aagcggaatg tgaaagccca gggctcaacc

601 ctggaagtgc atcccgaact gcgtcgcttg aatctcggag ggggacagag aattcccggt

661 gtagaggtga aattcgtaga tatcgggagg aataccactg gcgaaggcgc tgtcctggac

721 gaagattgac gctgaggtgc gaaagcgtgg ggagcaaaca ggattagata ccctggtagt

781 ccacgctgta aacgatgagt gctggacggt ggaggatttg accccttcgc tgtcgaagct

841 aacgcgttaa gcactccgcc tggggagtac ggtcgcaaga ctaaaactca aaggaattga

901 cgggggcccg cacaagcggt ggagcatgtg gtttaattcg acgcaacgcg cagaacctta

961 cctgggttaa atccactgga acctggctga aaggctgggg tgcccttcgg ggagccggtg

1021 agaaggtgct gcatggctgt cgtcagctcg tgtcgtgaga tgttgggtta agtcccgcaa

1081 cgagcgcaac ccctatcgcc agttgccacc attgagttgg gaactctggc gagactgccg

1141 gtctaaaccg gaggaaggtg gggacgacgt caagtcctca tggccctcat gcccagggct

1201 acacacgtgc tacaatggct ggtacaaaga gtcgcaagct cgcgagagta agcaaatctc

1261 aaaaaaccag tctcagttcg gattgcagtc tgcaactcga ctgcatgaag ctggaatcgc

1321 tagtaatcgg agatcagcac gctccggtga atacgttccc gggccttgta cacaccgccc

1381 gtcacaccat gggagtcggc tgctccagaa gtaggaacct caaccgcaag gaaaggccct

1441 accaaggagc ggtcggtgac tggggtgaag tcgtaacaag gtagccgtag gggaacctgc

1501 ggctggatca cctc

>*Nannocystis exedens* DSM 71

1 tgatcctggc tcagagcgaa cgtttgcggc gggcctaaca catgcaagtc gaacgggcta

61 gcaatagtca gtggcgcacg ggtgcgtaac acgtaggtaa tcaacccctc ggttcgggat

121 aacgttctga aaggagcgct aataccggac gtgtcttcgg gagcttcggc tcctgtcgag

181 aaagacccgc aagggttgcc gagggacgac tgcggcccat cagctagttg gcgaggtaat

241 agctcaccaa ggcgaabacg ggtagctggt ctgagaggat gatcagtcac actggaactg

301 agacacggtc cagactccta cgggaggcag cagtggggaa tattgcgcaa tgggcgaaag

361 cctgacgcag ccacgccgcg tgagcgatga aggccttcgg gtcgtaaagc tctgtgggga

421 gagacgaaga aagcctgtga agagcaggcc ttgacggtat ctccttagca agcaccggct

481 aactccgtgc cagcagccgc ggtaatacgg agggtgcgaa cgttgctcgg aatcattggg

541 cgtaaagcgc acgtaggcgg cggcgtaagc gggatgtgaa agcccagggc tcaaccctgg

601 aagtgcatcc cgaactgcgt cgcttgaatc tcggaggggg acagagaatt cccggtgtag

661 aggtgaaatt cgtagatatc gggaggaata ccagtggcga aggcgctgtc ctggacgaag

721 attgacgctg aggtgcgaaa gcgtggggag caaacaggat tagataccct ggtagtccac

781 gctgtaaacg atgagtgctg gacggtggag gatttgaccc cttcgctgtc gaagctaacg

841 cgttaagcac tccgcctggg gagtacggtc gcaagactaa aactcaaagg aattgacggg

901 ggcccgcaca agcggtggag catgtggttt aattcgacgc aacgcgcaga accttacctg

961 ggttaaatcc actggaacct ggctgaaagg ctggggtgcc cttcggggag ccggtgagaa

1021 ggtgctgcat ggctgtcgtc agctcgtgtc gtgagatgtt gggttaagtc ccgcaacgag

1081 cgcaacccct atcgccagtt gccaccattg agttgggaac tctggcgaga ctgccggtct

1141 aaaccggagg aaggtgggga cgacgtcaag tcctcatggc ccttatgccc agggctacac

1201 acgtgctaca atggctggta caaagagccg caagcccgcg agggtgagca aatctcaaaa

1261 aaccagtctc agttcggatt gcagtctgca actcgactgc atgaagctgg aatcgctagt

1321 aatcggagat cagcacgctc cggtgaatac gttcccgggc cttgtacaca ccgcccgtca

1381 caccatggga gtcggctgct ccagaagtag gaacctcaac cgcaaggaaa ggccctacca

1441 aggagcggtc ggtgactggg gtgaagtcgt aacaaggtaa

>*Haliangium ochraceum* DSM14365

1 tttgatcctg gctcagagcg aacgttagcg gcgggcttaa cacatgcaag tcgagcgaga

61 aaggagactt cggtctccga gtaaagcggc ggacgggtga gtaacacgtg ggtaacctgc

121 ctttcagtgg gggacaacta cccgaaaggg tggctaatac cgcatacgtc atacgggacc

181 gaggtcctgt atgggaaagc tagccaatac ttgtaagctg gcgctgaaag aggggcccgc

241 gtcgcatcag ctagttggtg aggtaacggc tcaccaaggc aaagacgcgt agctggtctg

301 agaggatgat cagccacact gggactgaga cacggcccag actcctacgg gaggcagcag

361 tggggaatat tggacaatgg gcgaaagcct gatccagcca cgccgcgtga gtgatgaagg

421 ccctagggtc gtaaagctct gtggagagag acgaaaccta ccaggttaag agctgggtag

481 ttgacggtat ctctttagca agcaccggct aactccgtgc cagcagccgc ggtaatacgg

541 agggtgcaaa cgttgctcgg aattattggg cgtaaagcgc acgtaggcgg cgcgataagt

601 cggatgtgaa agccctgggc ttaacctagg aagtgcattc gaaactgtcg cgcttgagta

661 cgggagaggg ttgcggaatt cccggtgtag aggtgaaatt cgtagatatc gggaggaaca

721 ccagtggcga aggcggcagc ctggaccgat actgacgctg aggtgcgaaa gcgtggggag

781 caaacaggat tagataccct ggtagtccac gccgtaaacg atgagtgcta gatgcagcgg

841 gtattgaccc ctgctgcgtc gcagctaacg cattaagcac tccgcctggg gagtacggcc

901 gcaaggctaa aactcaaagg aattgacggg ggcccgcaca agcggtggag catgtggttt

961 aattcgacgc aacgcgcaga accttacctg ggttaaatcc accagaatcc cctagagata

1021 ggggagtgcc ttcggggaat tggtgagaag gtgctgcatg gctgtcgtca gctcgtgtcg

1081 tgagatgttg ggttaagtcc cgcaacgagc gcaacctctg tcgtcagttg ccaacgggta

1141 agccgggcac tctgacgaga ctgccgacgt taagtcggag gaaggtggag atgacgtcaa

1201 gtcctcatgg cccttatgcc cagggctaca cacgtgctac aatggtcggt acaaagagca

1261 gcgatatcgt gaggttaagc taatctcaaa aaaccggcct cagttcggat tgaagtctgc

1321 aactcgactt catgaaggtg gaatcgctag taatcgtaga tcagcacgct acggtgaata

1381 cgttcccggg ccttgtacac accgcccgtc acaccatgga agtcagttgc tctagaagtc

1441 ggtgagctaa cctcgtgagg cagccgccta aagagtgact ggtaactggg gtgaagtcgt

1501 aacaagg

>*Haliangium tepidum* SMP-10

1 gatcctggct cagagcgaac gttagcggcg ggcttaacac atgcaagtcg agcgagaaag

61 gcggcttcgg ctgccgagta cagcggcgga cgggtgagta acacgtgggt aacctgccct

121 tcagtggggg acaaccaccc gaaagggtgg ctaataccgc atacgtcatc gggtgctgcg

181 gcaccggatg ggaaagctag cctctgcaag caagctggcg ctgaaggagg ggcccgcgtc

241 gcatcagcta gttggtgggg taatggctca ccaaggcaaa gacgcgtagc tggtctgaga

301 ggatgatcag ccacactggg actgagacac ggcccagact cctacgggag gcagcagtgg

361 ggaatattgg acaatgggcg caagcctgat ccagccacgc cgcgtgagtg atgaaggccc

421 tcgggtcgta aagctctgtg gggagggacg aagcctggcg ggtgaatagn ccggcagatg

481 acggtacctc cttagcaagc accggctaac tccgtgccag cagccgcggt aatacggagg

541 gtgcaaacgt tgctcggaat tactgggcgt aaagcgcacg taggcggcct gataagtcgg

601 atgtgaaagc cccgggctca acctgggaag tgcattcgaa actgttgggc ttgagtacgg

661 gagagggttg cggaattccc ggtgtagagg tgaaattcgt agatatcggg aggaacacca

721 gtggcgaagg cggcagcctg gaccgatact gacgctgagg tgcgaaagcg tggggagcaa

781 acaggattag ataccctggt agtccacgcc gtaaacgatg agtgctagat gcagcgggta

841 ttgacccctg ctgcgtcgta gctaacgcgt taagcactcc gcctggggag tacggccgca

901 aggctaaaac tcaaaggaat tgacgggggc ccgcacaagc ggtggagcat gtggttcaat

961 tcgacgcaac gcgcagaacc ttacctgggt taaatccgcc ggaatcccct agagataggg

1021 gagtgccctt cggggaatcg gtgagaaggt gctgcatggc tgtcgtcagc tcgtgtcgtg

1081 agatgttggg ttaagtcccg caacgagcgc aacctctgtc gtcagttgcc aacggggaag

1141 ccgggcactc tgacgagact gccgacgtta agtcggagga aggtggagat gacgtcaagt

1201 cctcatggcc cttatgccca gggctacaca cgtgctacaa tggtcggtac aaagagcagc

1261 gaagccgcga ggtggagcca atctcaaaaa accggcctca gttcggattg gagtctgcaa

1321 ctcgactcca tgaaggtgga atcgctagta atcgtagatc agcacgctac ggtgaatacg

1381 ttcccgggcc ttgtacacac cgcccgtcac accatgggag tcagttgctc tagaagtcgg

1441 tgagctaacc aagtgaggca gccgcctaaa gagtgactgg taactggggt gaagtcgtaa

1501 caaggtaa

>*Enhygromyxa salina* SWB004

1 tgcagtcgnc gagncctctt cggaggggac agtggcgcac gggtgcgtaa cacgtggata

61 accaaccccc tggttcggga caacactggg aaaccggtgc taataccggc tatggcgcat

121 ggatcatcag ntctgtgcga gaaaggcccg caagggtcgc caggggacgg gtccgcgtcc

181 catcagcttg ttggtgaggt aatggctcac caaggcaaag acgggtagct ggtctgagag

241 gatgatcagc cacactggaa ctgagacacg gtccagactc ctacgggagg cagcagtggg

301 gaatattgcg caatgggcga aagcctgacg cagccacgcc gcgtgagcga tgaaggtcct

361 aggatcgtaa agctctgtgg agggagaaga ataaagccgg tgaagagccg gccctgacgg

421 tatcccttta gcaagcaccg gctaactccg tgccagcagc cgcggtaata cggagggtgc

481 aaacgttgct cggaatcatt gggcgtaaag cgcgcgtagg cggcttggca agttggatgt

541 gaaagccctg ggctcaaccc aggaggtgca tacaaaactg ccaggcttga atctcggagg

601 gggtcaggga attcccggtg tagaggtgaa attcgtagat atcgggagga acaccagtgg

661 cgaaggcgct gacctggacg atgattgacg ctgaggcgcg aaagcgtggg gagcaaacag

721 gattagatac cctggtagtc cacgccgtaa acgatgagtg ctaggcgccg gcggattgac

781 cccgtcggtg ccgaagctaa cgcgttaagc actccgcctg gggagtacgg tcgcaagact

841 aaaactcaaa ggaattgacg gggcccgca caagcggtgg agcatgtggt ttaattcgac

901 gctacgcgca gaaccttacc tgggttaaat ccaccagaat ccggcccaaa agctggagtg

961 cccttcgggg aattggtgag aaggtgctgc atggctgtcg tcagctcgtg tcgtgagatg

1021 ttgggttaag tcccgcaacg agcgcaaccc ctgtcgtctg ttgccatcat ttagttggga

1081 actcagacgg tactgccggc ctaaaccgga ggaaggtggg gatgacgtca agtcctcatg

1141 gccctcatgc ccagggctac acacgtgcta caatggctgg tacaaagagc cgctaactcg

1201 cgagagtacg ctaatctcat aaaaccagtc tcagttcgga ttggagtctg caactcgact

1261 ccatgaagct ggaatcgcta gtaatcggag atcagcacgc tccggtgaat acgttcccgg

1321 gccttgtaca caccgcccgt cacaccatgg gagtcgattg ctccagaagt gggtagccta

1381 accttcggga gggcgctnac caaggagtga tcgggactgg gnaa

>*Enhygromyxa salina* SWB005

1 tcnngctccg gccgcctggc cgcgggntta gagtttgatc ctggctcaga gcgaacgttt

61 gcggcgggcc taacacatgc aagtcgaacg agaatctctt cggagaggac agtggcgcac

121 gggtgcgtaa cacgtggata accaaccccc tggttcggga caacactggg aaaccggtgc

181 taataccgta tgtgacgcac agatcgtcag gtttgtgcga gaaaggcacg caagggtcgc

241 caggggacgg gtccgcgtcc catcagcttg ttggtgaggt aatggctcac caaggctaag

301 acgggtagct ggtctgagag gatgatcagc cacactggaa ctgagacacg gtccagactc

361 ctacgggagg cagcagtggg gaatattgcg caatgggcga aagcctgacg cagccacgcc

421 gcgtgagcga tgaaggtcct aggatcgtaa agctctgtgg agggagaaga acaaagccgg

481 tgaagagccg gccctgacgg tatcccttta gcaagcaccg gctaactccg tgccagcagc

541 cgcggtaata cggagggtgc aaacgttgct cggaattatt gggcgtaaag cgcgcgtagg

601 cggctaggca agttggatgt gaaagccctg ggctcaaccc aggaggtgca ttcaaaactg

661 cctggcttga atctcggagg gggtcaggga attcccggtg tagaggtgaa attcgtagat

721 atcgggagga acaccagtgg cgaaggcgct gacctggacg atgattgacg ctgaggcgcg

781 aaagcgtggg gagcaaacag gattagatac cctggtagtc cacgccgtaa acgatgagtg

841 ctaggcgccg gcggattgac cccgtcggtg ccgaagctaa cgcgttaagc actccgcctg

901 gggagtacgg tcgcaagact aaaactcaaa ggaattgacg gggcccgca caagcggtgg

961 agcatgtggt ttaattcgac gctacgcgca gaaccttacc tgggttaaat ccaccagaat

1021 ccggcccaaa agctggagtg cccttcgggg aattggtgcg aaggtgctgc atggctgtcg

1081 tcagctcgtg tcgtgagatg ttgggttaag tcccgcaacg agcgcaaccc ctgtcgtctg

1141 ttgccatcat tcagttggga actcagacgg tactgccggc ctaaaccgga ggaaggtggg

1201 gatgacgtca agtcctcatg gccctcatgc ccagggctac acacgtgcta caatggctgg

1261 tacaaagagc cgcgaactcg cgagagtaag ctaatctcat aaaaccagtc tcagttcgga

1321 ttggagtctg caactcgact ccatgaagct ggaatcgcta gtaatcggag atcagcacgc

1381 tccggtgaat acgttcccgg gccttgtaca caccgcccgt cacaccatgg gagtcgattg

1441 ctccagaagt gggtagccta accttcggga gggcgcttac caaggagtga tcggtgactg

1501 gggtgaagtc gtaacaaggt atccctaccg gaaggtgggg ctggatcacctc

>*Enhygromyxa salina* SWB006

1 gtttgatcct ggctcagagc ggacgtttgc ggcgggctta acacatgcaa gtcgaacgag

61 aacctcttcg gaggggacag tggcgcacgg gtgcgtaaca cgtggataac caaccccctg

121 gttcgggaca acactgggaa accggtgcta ataccggcta tggcgcatgg atcatcaggt

181 ctgtgcgaga aaggcccgca agggtcgcca ggggacgggt ccgcgtccca tcagcttgtt

241 ggtgaggtaa tggctcacca aggcgaagac gggtagctgg tctgagagga tgatcagcca

301 cactggaact gagacacggt ccagactcct acgggaggca gcagtgggga atattgcgca

361 atgggcgaga gcctgacgca gccacgccgc gtgagcgatg aaggtcctag gatcgtaaag

421 ctctgtggag ggagaagaat aaagccggtg aagagccggc cttgacggta tccctttagc

481 aagcaccggc taactccgtg ccagcagccg cggtaatacg gagggtgcaa acgttgctcg

541 gaatcattgg gcgtaaagcg cgcgtaggcg gctcggcaag ttggatgtga aagccctggg

601 ctcaacccag gaggtgcata caaaactgcc aggcttgaat ctcggagggg gtcagggaat

661 tcccggtgta gaggtgaaat tcgtagatat cgggaggaac accagtggcg aaggcgctga

721 cctggacgat gattgacgct gaggcgcgaa agcgtgggga gcgaacagga ttagataccc

781 tggtagtcca cgccgtaaac gatgagtgct aggcgccggc ggattgaccc cgtcggtgcc

841 gaagctaacg cgttaagcac tccgcctggg gagtacggtc gcaagactaa aactcaaagg

901 aattgacggg ggcccgcaca agcggtggag catgtggttt aattcgacgc tacgcgcaga

961 accttacctg ggttaaatcc accagaatcc ggcccaaaag ctggagtgcc cttcggggaa

1021 ttggtgagaa ggtgctgcat ggctgtcgtc agctcgtgtc gtgagatgtt gggttaagtc

1081 ccgcaacgag cgcaacccct gtcgtctgtt gccatcattt agttgggaac tcagacggta

1141 ctgccggcct aaaccggagg aaggtgggga tgacgtcaag tcctcatggc cctcatgccc

1201 agggctacac acgtgctaca atggctggta caaagagccg ctaactcgcg agagtacgct

1261 aatctcataa aaccagtctc agttcggatt ggagtctgca actcgactcc atgaagctgg

1321 aatcgctagt aatcggagat cagcacgctc cggtgaatac gttcccgggc cttgtacaca

1381 ccgcccgtca caccatggga gtcgattgct ccagaagtgg gtagcctaac cttcgggagg

1441 gcgcttacca aggagtgatc ggtggctggg gtgaagtcgt aacaaggtat ccctaccgga

1501 aggtggggct ggatcacctc

>*Enhygromyxa salina* SWB007

1 tgcagtcgnc gagacctctt cggagggnnn gtggcgcacg ggtgcgtaac acgtggataa

61 ccaaccccct ggttcgggac aacactggga aaccggtgct aataccggct atggcgcatg

121 gatcatcagg tctgtgcgag aaaggcccgc aagggtcgcc aggggacggg tccgcgtccc

181 atcagcttgt tggtgaggta atggctcacc aaggcgaaga cgggtagctg gtctgagagg

241 atgatcagcc acactggaac tgagacacgg tccagactcc tacgggaggc agcagtgggg

301 aatattgcgc aatgggcgaa agcctgacgc agccacgccg cgtgagcgat gaaggtccta

361 ggatcgtaaa gctctgtgga gggagaagaa taaagccggt gaagagccgg ccttgacggt

421 atccctttag caagcaccgg ctaactccgt gccagcagcc gcggtaatac ggagggtgca

481 aacgttgctc ggaatcattg ggcgtaaagc gcgcgtaggc ggcttggcaa gttggatgtg

541 aaagccctgg gctcaaccca ggaggtgcat acaaaactgc caggcttgaa tctcggaggg

601 ggtcagggaa ttcccggtgt agaggtgaaa ttcgtagata tcgggaggaa caccagtggc

661 gaaggcgctg acctggacga tgattgacgc tgaggcgcga aagcgtgggg agcaaacagg

721 attagatacc ctggtagtcc acgccgtaaa cgatgagtgc taggcgccgg cggattgacc

781 ccgtcggtgc cgaagctaac gcgttaagca ctccgcctgg ggagtacggt cgcaagacta

841 aaactcaaag gaattgacgg gggcccgcac aagcggtgga gcatgtggtt taattcgacg

901 ctacgcgcag aaccttacct gggttaaatc caccagaatc cggcccaaaa gctggagtgc

961 ccttcgggga attggtgaga aggtgctgca tggctgtcgt cagctcgtgt cgtgagatgt

1021 tgggttaagt cccgcaacga gcgcaacccc tgtcgtctgt tgccatcatt tagttgggaa

1081 ctcagacggt actgccggcc taaaccggag gaaggtgggg atgacgtcaa gtcctcatgg

1141 ccctcatgcc cagggctaca cacgtgctac aatggctggt acaaagagcc gctaactcgc

1201 gagagtacgc taatctcata aaaccagtct cagttcggat tggagtctgc aactcgactc

1261 catgaagctg gaatcgctag taatcggaga tcagcacgct ccggtgaata cgttcccggg

1321 ccttgtacac accgcccgtc acaccatggg agtcgattgc tccagaagtg ggtagcctaa

1381 ccttcgggag ggcgcntacc aaggagtgat cggnnc

>*Enhygromyxa salina* SHK1T

1 gatcctggct cagagcgaac gtttgcggcg ggcttaacac atgcaagtcg aacgagaacc

61 tcttcggagg ggacagtggc gcacgggtgc gtaacacgtg gataaccaac cccctggttc

121 gggacaacac tgggaaaccg gtgctaatac cggctatggc gcatgagtca caaggcttgt

181 gcgagaaagg cccgcaaggg tcgccagggg acgggtccgc gtcccatcag cttgttggtg

241 aggtaatggc tcaccaaggc gaagacgggt agctggtctg agaggatgat cagccacact

301 ggaactgaga cacggtccag actcctacgg gaggcagcag tggggaatat tgcgcaatgg

361 gcgaaagcct gacgcagcca cgccgcgtga gcgatgaagg tcctaggatc gtaaagctct

421 gtggagggag aagaataaag ccggtgaaga gccggccttg acggtatccc tttagcaagc

481 accggctaac tccgtgccag cagccgcggt aatacggagg gtgcaaacgt tgctcggaat

541 cattgggcgt aaagcgcgcg taggcggctt ggcaagttgg atgtgaaagc cctgggctca

601 acccaggagg tgcatacaaa actgccaggc ttgaatctcg gaggggggtca gggaattccc

661 ggtgtagagg tgaaattcgt agatatcggg aggaacacca gtggcgaagg cgctgacctg

721 gacgatgatt gacgctgagg cgcgaaagcg tggggagcaa acaggattag ataccctggt

781 agtccacgcc gtaaacgatg agtgctaggc gccggcggat tgaccccgtc ggtgccgaag

841 ctaacgcgtt aagcactccg cctggggagt acggtcgcaa gactaaaact caaaggaatt

901 gacgggggcc cgcacaagcg gtggagcatg tggtttaatt cgacgctacg cgcagaacct

961 tacctgggtt aaatccacca gaatccggcc caaaagctgg agtgcccttc ggggaattgg

1021 tgagaaggtg ctgcatggct gtcgtcagct cgtgtcgtga gatgttgggt taagtcccgc

1081 aacgagcgca acccctgtcg tctgttgcca tcatttagtt gggaactcag acggtactgc

1141 cggcctaaac cggaggaagg tggggatgac gtcaagtcct catggccctc atgcccaggg

1201 ctacacacgt gctacaatgg ctggtacaaa gagccgctaa ctcgcgagag tacgctaatc

1261 tcataaaacc agtctcagtt cggattggag tctgcaactc gactccatga agctggaatc

1321 gctagtaatc ggagatcagc acgctccggt gaatacgttc ccgggccttg tacacaccgc

1381 ccgtcacacc atgggagtcg attgctccag aagtgggtag cctaaccttc gggagggcgc

1441 ttaccaagga gtgatcggtg actggggtga agtcgtaaca

>*Enhygromyxa salina* SMK13

1 atcctggctc agagcgaacg tttgcggcgg gcttaacaca tgcaagtcga acgagaacct

61 cttcggaggg gacagtggcg cacgggtgcg taacacgtgg ataaccaacc ccctggttcg

121 ggacaacact gggaaaccgg tgctaatacc ggctatggcg catgagtcac aaggcttgtg

181 cgagaaaggc ccgcaagggt cgccagggga cgggtccgcg tcccatcagc ttgttggtga

241 ggtaatggct caccaaggcg aagacgggta gctggtctga gaggatgatc agccacactg

301 gaactgagac acggtccaga ctcctacggg aggcagcagt ggggaatatt gcgcaatggg

361 cgaaagcctg acgcagccac gccgcgtgag cgatgaaggt cctaggatcg taaagctctg

421 tggagggaga agaataaagc cggtgaagag ccggccttga cggtatccct ttagcaagca

481 ccggctaact ccgtgccagc agccgcggta atacggaggg tgcaaacgtt gctcggaatc

541 attgggcgta aagcgcgcgt aggcggcttg gcaagttgga tgtgaaagcc ctgggctcaa

601 cccaggaggt gcatacaaaa ctgccaggct tgaatctcgg agggggtcag ggaattcccg

661 gtgtagaggt gaaattcgta gatatcggga ggaacaccag tggcgaaggc gctgacctgg

721 acgatgattg acgctgaggc gcgaaagcgt ggggagcaaa caggattaga taccctggta

781 gtccacgccg taaacgatga gtgctaggcg ccggcggatt gaccccgtcg gtgccgaagc

841 taacgcgtta agcactccgc ctggggagta cggtcgcaag actaaaactc aaaggaattg

901 acgggggccc gcacaagcgg tggagcatgt ggtttaattc gacgctacgc gcagaacctt

961 acctgggtta aatccaccag aatccggccc aaaagctgga gtgcccttcg gggaattggt

1021 gagaaggtgc tgcatggctg tcgtcagctc gtgtcgtgag atgttgggtt aagtcccgca

1081 acgagcgcaa cccctgtcgt ctgttgccat catttagttg ggaactcaga cggtactgcc

1141 ggcctaaacc ggaggaaggt ggggatgacg tcaagtcctc atggccctca tgcccagggc

1201 tacacacgtg ctacaatggc tggtacaaag agccgctaac tcgcgagagt gcgctaatct

1261 cataaaacca gtctcagttc ggattggagt ctgcaactcg actccatgaa gctggaatcg

1321 ctagtaatcg gagatcagca cgctccggtg aatacgttcc cgggccttgt acacaccgcc

1381 cgtcacacca tgggagtcga ttgctccaga agtgggtagc ctaaccttcg ggagggcgct

1441 taccaaggag tgatcggtga ctggggtgaa gtcgtaaca

>*Plesiocystis pacifica* SIR1^T

1 atcctggctc agagcgaacg tttgcggcgg gcctaacaca tgcaagtcga acgagaagcc

61 cttcggggtg gaaagtggcg cacgggtgcg taacacgtgg ataaccaacc ccccagtttg

121 ggacaacacc gggaaactgg tgctaatacc agatgtgtcg cgggagtcgt caggctccct

181 cgagaaaggc acgcaagggt cgctggggga cgggtccgcg tcccatcagc tagttggtga

241 ggtaatggct caccaaggct aagacgggta gctggtctga gaggatgatc agccacactg

301 gaactgagac acggtccaga ctcctacggg aggcagcagt ggggaatatt gcgcaatggg

361 cgaaagcctg acgcagccac gccgcgtgag cgatgaaggt tttcggatcg taaagctctg

421 tggagggaga agaataaagt cagtgaagag ctggccttga cggtatccct ttagcaagca

481 ccggctaact ccgtgccagc agccgcggta atacggaggg tgcaaacgtt gctcggaatc

541 attgggcgta aagcgcgcgt aggcggcttg gcaagttgga tgtgaaagcc ctgggctcaa

601 cccaggaagt gcatccaaaa ctgcctggct tgaatctcgg agggggtcag ggaattctcg

661 gtgtagaggt gaaattcgta gatatcggga ggaacaccag tggcgaaggc gctgacctgg

721 acgatgattg acgctgaggt gcgaaagcat ggggagcgaa caggattaga taccctggta

781 gtccatgccg taaacgatga gtgctaggcg ccggcggatg accccgtcgg tgccgaagct

841 aacgcgttaa gcactccgcc tggggagtac ggtcgcaaga ctaaaactca aaggaattga

901 cgggggcccg cacaagcggt ggagcatgtg gtttaattcg acgcaacgcg cagaacctta

961 cctgggttaa gtccacgaga atccggccca aaagctggag tgcccttcgg ggaattcgtg

1021 cgaaggtgct gcatggctgt cgtcagctcg tgtcgtgaga tgttgggtta agtcccgcaa

1081 cgagcgcaac ccctgccgtc tgttgccatc atttagttgg gaactcagac ggtactgccg

1141 gcctaaaccg gaggaaggtg gggatgacgt caagtcctca tggccctcat gcccagggct

1201 acacacgtgc tacaatggtt ggtacaaaga gtcgctagcc cgcgaggga cgctaacctc

1261 aaaaaaccaa tctcagttcg gattggagtc tgcaactcga ctccatgaag ctggaatcgc

1321 tagtaatcgg agatcagcac gctccggtga atacgttccc gggccttgta cacaccgccc

1381 gtcacaccat gggagtcgat tgctccagaa gtgggtagcc taaccttcgg gagggcgctt

1441 accaaggagt gatcggtgac tggggtgaag tcgtaacaag gtaa

>*Plesiocystis pacifica* SHI-1

1 tgatcctggc tcagagcgaa cgtttgcggc gggcctaaca catgcaagtc gaacgagaaa

61 gccttcgggg tggaaagtgg cgcacgggtg cgtaacacgt ggataaccaa ccccccagtt

121 tgggacaaca ccgggaaact ggtgctaata ccagatgtgt cgcgggagtc gtcaggctcc

181 ctcganaaag gcacgcaagg gtcgctgggg gacgggtccg cgtcccatca gctagttggt

241 gaggtaatgg ctcaccaagg ctaagacggg tagctggtct gagaggatga tcagccacac

301 tggaactgag acacggtcca gactcctacg ggaggcagca gtggggaata ttgcgcaatg

361 ggcgaaagcc tgacgcagcc acgccgcgtg agcgatgaag gttttcggat cgtaaagctc

421 tgtggaggga gaagaataaa gtcagtgaag agctggcctt gacggtatcc ctttcagcaa

481 gcaccggcta actccgtgcc agcagccgcg gtaatacgga gggtgcaaac gttgctcgga

541 atcattgggc gtaaagcgcg cgtaggcggc ttggcaagtt ggatgtgaaa gccctgggct

601 caacccagga agtgcatcca aaactgcctg gcttgaatct cggagggggt cagggaattc

661 tcggtgtaga ggtgaaattc gtagatatcg ggaggaacac cagtggcgaa ggcgctgacc

721 tggacgatga ttgacgctga ggtgcgaaag catggggagc gaacaggatt agataccctg

781 gtagtccatg ccgtaaacga tgagtgctag gcgccggcgg atgaccccgt cggtgccgaa

841 gctaacgcgt taagcactcc gcctggggag tacggtcgca agactaaaac tcaaaggaat

901 tgacgggggc ccgcacaagc ggtggagcat gtggtttaat tcgacgcaac gcgcagaacc

961 ttacctgggt taaatccacg agaatccggc ccaaaagctg gagtgcttcg gggaattcgt

1021 gcgaaggtgc tgcatggctg tcgtcagctc gtgtcgtgag atgttgggtt aagtcccgca

1081 acgagcgcaa cccctgccgt ctgttgccat catttagttg ggaactcaga cggtactgcc

1141 ggcctaaacc ggaggaaggt ggggatgacg tcaagtcctc atggccctca tgcccagggc

1201 tacacacgtg ctacaatggt tggtacaaag agtcgctagc ccgcgaggga cgctaacctc

1261 aaaaaaccaa tctcagttcg gattggagtc tgcaactcga ctccatgaag ctggaatcgc

1321 tagtaatcgg agatcagcac gctccggtga atacgttccc gggccttgta cacaccgccc

1381 gtcacaccat gggagtcgat tgctccagaa gtgggtagcc taaccttcgg gagggcgctt

1441 accaaggagt gatcggtgac tggggtgaag tcgtaacaag gta

>Myxobacterium SMH-27-4 (*Paraliomyxa miuraensis*)

1 gagtttgatc ctggctcaga gcgaacgttt gcggcgggcc taacacatgc aagtcgaacg

61 ataactcggg cttcggcctg ggggaaagtg gcgcacgggt gcgtaacacg tgggtaatca

121 accctttgga ctgggacaac ccggggaaac tcgggctaat accggatgtg tcgtgggagt

181 cttcgggcac ccacgggaaa ggcccgcaag ggctaccaaa ggacgagctc gcggcccatc

241 agcttgttgg tgaggtaatg gctcaccaag gctaagacgg gtagctggtc tgagaggatg

301 atcagccaca ctggaactga gacacggtcc agactcctac gggaggcagc agtggggaat

361 attgcgcaat gggcgaaagc ctgacgcagc cacgccgcgt gtgcgatgaa ggtcttcgga

421 tcgtaaagca ctgtgggggg agaagaacaa agccggtgaa gagccggccc tgacggtatc

481 cccttagcaa gcaccggcta actccgtgcc agcagccgcg gtaatacgga gggtgcaaac

541 gttgctcgga atcactgggc gtaaagcgca cgtaggcggc cgtgtaagtt ggatgtgaaa

601 gccctgggct caacccagga agtgcatcca aaactgtgcg gcttgagtac cggaggggct

661 cagagaattc ccggtgtaga ggtgaaattc gtagatatcg ggaggaatac cagtggcgaa

721 ggcgctgagc tggacggtaa ctgacgctga ggtgcgaaag cgtgggtagc aaacaggatt

781 agataccctg gtagtccacg ccgtaaacga tgagtgctag gcggtgggag attttgaccc

841 tctcgctgtc gaagctaacg cgttaagcac tccgcctggg gagtacggtc gcaagactaa

901 aactcaaagg aattgacggg ggcccgcaca agcggtggag catgtggttt aattcgacgc

961 aacgcgcaga accttacctg ggttaaatcc agcggaatcc ggccgaaatg ctggagtgcc

1021 cttcggggaa tcgctgcgaa ggtgctgcat ggctgtcgtc agctcgtgtc gtgagatgtt

1081 gggttaagtc ccgcaacgag cgcaacccct atcgccagtt gccatcattc agttgggaac

1141 tctggcgaga ctgccggcca aaaccggagg aaggtgggga tgacgtcaag tcctcatggc

1201 ccttatgccc agggctacac acgtgctaca atggctggta caatgagccg cgaacccgtg

1261 agggcaagct aatctcaaaa aaccagtctc agttcggatt ggagtctgca actcgactcc

1321 atgaagctgg aatcgctagt aatcggagat cagcacgctc cggtgaatac gttcccgggc

1381 cttgtacaca ccgcccgtca caccatggga gtcggttgct ccagaagtag gtatcccaac

1441 ccgcaaggga cggagcttac caaggagtga tcggtgactg gggtgaagtc gtaacaaggt

1501 agcc

>*Pseudenhygromyxa salsuginis* SYR2T

1 agagtttgat cctggctcag agcgaacgtt tgcggcgggc ctaacacatg caagtcgaac

61 gagaatcccy tcggggagga aagtggcgca cgggtgcgta acacgtggat aaccaacccc

121 ctggctcggg acaacactgg gaaaccggtg ctaataccgg atatggcgag tggatcatca

181 ggtctgctcg agaaaggcac gcaagggtca ccaggggacg ggtccgcgtc ccatcagctt

241 gttggtgagg taatggctca ccaaggctaa gacgggtagc tggtctgaga ggatgatcag

301 ccacactgga actgagacac ggtccagact cctacgggag gcagcagtgg ggaatattgc

361 gcaatgggcg aaagcctgac gcagccacgc cgcgtgagcg atgaaggtct taggatcgta

421 aagctctgtg gggggagaag aacaaagccg gtgaagagcc ggccctgacg gtatcccctt

481 agcaagcacc ggctaactcc gtgccagcag ccgcggtaat acggagggtg caaacgttgc

541 tcggaatcat tgggcgtaaa gcgcgcgtag gcggcttggc aagttggatg tgaaagccct

601 gggctcaacc caggatgtgc attcaaaact gccwagcttg aatctcggag ggggacaggg

661 aattctcggt gtagaggtga aattcgtaga tatcgggagg aacaccagtg gcgaaggcgc

721 tgtcctggac gatgattgac gctgaggcgc gaaagcgtgg ggagcaaaca ggattagata

781 ccctggtagt ccacgccgta aacgatgagt gctaggcgcc ggcggattga ccccgtcggt

841 gccgaagcta acgcgttaag cactccgcct ggggagtacg gtcgcaagac taaaactcaa

901 aggaattgac gggggcccgc acaagcggtg gagcatgtgg tttaattcga cgcaacgcgc

961 agaaccttac ctgggttaaa tccaccagaa tctggcccaa aagccggagt gcccttcggg

1021 gaattggtga gaaggtgctg catggctgtc gtcagctcgt gtcgtgagat gttgggttaa

1081 gtcccgcaac gagcgcaacc cctaccgtct gttgccagca tttagttggg aactcagacg

1141 gcactgccgg cctaaaccgg aggaaggtgg ggatgacgtc aagtcctcat ggccctcatg

1201 cccagggcta cacacgtgct acaatggctg gtacaaagag atgcgagccc gcgaggggaa

1261 gctaacctca aaaaaccagt ctcagttcgg attggagtct gcaactcgac tccatgaagc

1321 tggaatcgct agtaatcgga gatcagcacg ctccggtgaa tacgttcccg ggccttgtac

1381 acaccgcccg t

>*Myxococcus xanthus* DK1622

1 caattggaga gtttgatcct ggctcagaac gaacgctggc ggcgtgccta acacatgcaa

61 gtcgagcgcg aataggggca acccttagta gagcggcgca cgggtgcgta acacgtggat

121 aatctgcctg agtgctcggg ataaccagtc gaaagattgg ctaataccgg ataagcccac

181 ggtttcttcg gagactgagg gaaaaggtgg cctctgtata caagctatca cattcagatg

241 agtccgcggc ccatcagcta gttggcgggg taatggccca ccaaggcaac gacgggtagc

301 tggtctgaga ggacgatcag ccacactgga actgagacac ggtccagact cctacgggag

361 gcagcagtgg ggaattttgc gcaatgggcg aaagcctgac gcagcaacgc cgcgtgtgtg

421 atgaaggtct ttggattgta aagcactttc gaccgggaag aaaacccgtt ggctaacatc

481 caacggcttg acggtaccgg gagaagaagc accggctaac tctgtgccag cagccgcggt

541 aatacagagg gtgcaagcgt tgttcggaat tattgggcgt aaagcgcgtg taggcggcgt

601 gacaagtcgg gtgtgaaagc cctcagctca actgaggaag tgcgcccgaa actgttgtgc

661 ttgagtgccg gagagggtgg cggaattccc caagtagagg tgaaattcgt agatatgggg

721 aggaacaccg gtggcgaagg cggccacctg gacggtaact gacgctgaga cgcgaaagcg

781 tggggagcaa acaggattag ataccctggt agtccacgcc gtaaacgatg agaactaggt

841 gtcgtgggag ttgacccccg cggtgccgaa gctaacgcat taagttctcc gcctgggaag

901 tacggtcgca agactaaaac tcaaaggaat tgacgggggc ccgcacaagc ggtggagcat

961 gtggtttaat tcgacgcaac gcgcagaacc ttacctggtc ttgacatcct cagaatcctt

1021 cagagatgag ggagtgcccg caagggaact gagagacagg tgctgcatgg ctgtcgtcag

1081 ctcgtgtcgt gagatgttgg gttaagtccc gcaacgagcg caaccctcgc ctttagttgc

1141 cacgcaagtg gatctctaga gggactgccg gtgttaaacc ggaggaaggt ggggatgacg

1201 tcaagtcctc atggccttta tgaccagggc tacacacgtg ctacaatggc cggtacagag

1261 cgttgccaac ccgcgagggg gagctaatcg cataaaaccg gtctcagttc agattggagt

1321 ctgcaactcg actccatgaa ggaggaatcg ctagtaatcg cagatcagca cgctgcggtg

1381 aatacgttcc cgggccttgt acacaccgcc cgtcacacca tgggagtcga ttgctccaga

1441 aatcatctca ccaagaggtg ctcaaggagt ggtcggtaac tggggtgaag tcgtaacaag

1501 gtagccgtag gggaacctgc ggctggatca cctcctttct

>*Sorangium cellulosum* Soce56

1 ctacgaagtg acgtgggtcc caaaggcgag cgcaagctcg acctcgcacg tcataaagac

61 gagcaagtca ggcggtctta gccaccgtct gatgcaacag gatttaactg gagagtttga

121 tcctggctca gaacgaacgt tagcggcgcg cttaacacat gcaagtcgag cgagaaaggg

181 cttcggcccc ggtaaagcgg cgcacgggtg agtaacacgt aggtaatctg cccccaggtg

241 gtggataacg ttccgaaagg agcgctaata cagcatgaga ccacgccttc gaaaggaggt

301 gaggtcaaag ccggcctctt catgaaagct ggcgccaggg gatgagcctg cggcccatca

361 gctagttggt agggtaatgg cctaccaagg cgaagacggg tagctggtct gagaggatga

421 tcagccacac tggaactgag acacggtcca gactcctacg ggaggcagca gtggggaatc

481 ttgcgcaatg ggcgaaagcc tgacgcagcg acgccgcgtg agtgatgaag gccttcgggt

541 tgtaaagctc tgtggagggg gacgaataag ggttggctaa catccagctc gatgacggta

601 cccctttagc aagcaccggc taactctgtg ccagcagccg cggtaagaca gagggtgcaa

661 acgttgttcg gaattactgg gcgtaaagcg cgtgtaggcg gttcgtaaag tcaggtgtga

721 aagccctggg cttaacccag gaagtgcact tgaaactcac gaacttgagt cccggagagg

781 aaggcggaat tctcggtgta gaggtgaaat tcgtagatat cgagaggaac atcggtggcg

841 aaggcggcct tctggacggt gactgacgct gagacgcgaa agcgtgggga gcaaacagga

901 ttagataccc tggtagtcca cgccgtaaac gatgggtgct aggtgtcgcg ggctttgact

961 cctgcggtgc cgtagctaac gcattaagca ccccgcctgg ggagtacggc cgcaaggcta

1021 aaactcaaag gaattgacgg gggcccgcac aagcggtgga gcatgtggtt caattcgacg

1081 caacgcgcag aaccttacct gggctagaaa atgcaggaac ctggttgaaa gatcggggtg

1141 ctcttcggag aacctgtagt taggtgctgc atggctgtcg tcagctcgtg tcgtgagatg

1201 ttgggttaag tcccgcaacg agcgcaaccc ctatcgttag ttgccagcgg ttcggccggg

1261 cactctagcg agactgccga tatttaaatc ggaggaaggt ggggatgacg tcaagtcctc

1321 atggccctta tgtccagggc tacacacgtg ctacaatggg cggtacagac ggtcgcgaac

1381 ccgcgagggg aagccaatcc gaaaaaaccg tcctcagtac ggataagagt ctgcaactcg

1441 actctttgaa gttggaatcg ctagtaatcc ctgatcagca ggcaggggtg aatacgttcc

1501 cgggccttgt acacaccgcc cgtcacacca tgggagtcga ttgctccaga agtggctgcg

1561 ccaacccgca agggaggcag gcccccaagg agtggttggt aactggggtg aagtcgtaac

1621 aaggtagccg taggggaacc tgcggctgga tcacctcctt tctaaggaag cgcttaggcg

1681 ttacctgaac gtcaaaaccc acgcac

>*Escherichia albertii* DM104

1 cagtcgaacg gtaacaggaa gaagcttgct tctttgctga cgagtggcgg acgggtgagt

61 aatgtctggg aaactgcccg atggaggggg ataactactg gaaacggtag ctaataccgc

121 ataacgtcgc aagaccaaag agggggacct tcgggcctct tgccatcgga tgtgcccaga

181 tgggattagc ttgttggtga ggtaacggct caccaaggcg acgatcccta gctggtctga

241 gaggatgacc agccacactg gaactgagac acggtccaga ctcctacggg aggcagcagt

301 ggggaatatt gcacaatggg cgcaagcctg atgcagccat gccgcgtgta tgaagaaggc

361 cttcgggttg taaagtactt tcagcgggga ggaagggagt aaagttaata cctttgctca

421 ttgacgttac ccgcagaaga agcaccggct aactccgtgc cagcagccgc ggtaatacgg

481 agggtgcaag cgttaatcgg aattactggg cgtaaagcgc acgcaggcgg ttgattaagt

541 cagatgtgaa atccccgggc tcaacctggg aactgcatct gatactggtc agcttgagtc

601 tcgtagaggg gggtagaatt ccaggtgtag cggtgaaatg cgtagagatc tggaggaata

661 ccggtggcga aggcggcccc ctggacgaag actgacgctc aggtgcgaaa gcgtggggag

721 caaacaggat tagataccct ggtagtccac gccgtaaacg atgtcgactt ggaggttgtg

781 cccttgaggc gtggcttccg gagctaacgt gttaagtcga ccgcctgggg agtacggccg

841 caaggttaaa actcaaatga attgacgggg gcccgcacaa gcggtggagc atgtggttta

901 attcgatgca acgcgaagaa ccttacctgg tcttgacatc cacggaagtt ttcagagatg

961 agaatgtgcc ttcgggaacc gtgagacagg tgctgcatgg ctgtcgtcag ctcgtgttgt

1021 gaaatgttgg gttaagtccc gcaacgagcg caacccttat cctttgttgc cagcgattcg

1081 gtcgggaact caaaggagac tgccagtgat aaactggagg aaggtgggga tgacgtcaag

1141 tcatcatggc ccttacgacc agggctacac acgtgctaca atggcgcata caaagagaag

1201 cgacctcgcg agagcaagcg gacctcataa agtgcgtcgt agtccggatt ggagtctgca

1261 actcgactcc atgaagtcgg aatcgctagt aatcgtggat cagaatgcca cggtgaatac

1321 gttcccgggc cttgtacaca ccgcccgtca caccatggga gtgggttgca aaagaagtag

1381 gtagctta

>*Myxococcus fulvus* HW-1

1 tggagagttt gatcctggct cagaacgaac gctggcggcg tgcctaacac atgcaagtcg

61 agcgcgaata ggggcaaccc ttagtagagc ggcgcacggg tgcgtaacac gtggataatc

121 tgcctgagtg ctcgggataa ccagtcgaaa gattggctaa taccggataa gcccacggtt

181 tcttcggaga ctgagggaaa aggtggcctc tgtatacaag ctatcacatt cagatgagtc

241 cgcggcccat cagctagttg gcggggtaat ggcccaccaa ggcgacgacg ggtagctggt

301 ctgagaggac gatcagccac actggaactg agacacggtc cagactccta cgggaggcag

361 cagtggggaa ttttgcgcaa tgggcgaaag cctgacgcag caacgccgcg tgtgtgatga

421 aggtctttgg attgtaaagc actttcgacc gggaagaaaa cccgttggct aacatccaac

481 ggcttgacgg taccgggaga agaagcaccg gctaactctg tgccagcagc cgcggtaata

541 cagagggtgc aagcgttgtt cggaattatt gggcgtaaag cgcgtgtagg cggcgtgaca

601 agtcgggtgt gaaagccctc agctcaactg aggaagtgcg cccgaaactg tcgtgcttga

661 gtgccggaga gggtggcgga attccccaag tagaggtgaa attcgtagat atggggagga

721 acaccggtgg cgaaggcggc cacctggacg gtaactgacg ctgagacgcg aaagcgtggg

781 gagcaaacag gattagatac cctggtagtc cacgccgtaa acgatgagaa ctaggtgtcg

841 tgggagttga cccccgcggt gccgaagcta acgcattaag ttctccgcct gggaagtacg

901 gtcgcaagac taaaactcaa aggaattgac gggggcccgc acaagcggtg gagcatgtgg

961 tttaattcga cgcaacgcgc agaaccttac ctggtcttga catcctcgga atccttcaga

1021 gatgagggag tgcccgcaag ggaaccgaga gacaggtgct gcatggctgt cgtcagctcg

1081 tgtcgtgaga tgttgggtta agtcccgcaa cgagcgcaac cctcgccttt agttgccacg

1141 caagtggatc tctagaggga ctgccggtgt taaaccggag gaaggtgggg atgacgtcaa

1201 gtcctcatgg cctttatgac cagggctaca cacgtgctac aatggccggt acagagcgtt

1261 gccaacccgc gagggggagc taatcgcata aaaccggtct cagttcagat tggagtctgc

1321 aactcgactc catgaaggcg gaatcgctag taatcgcaga tcagcacgct gcggtgaata

1381 cgttcccggg ccttgtacac accgcccgtc acaccatggg agtcgattgc tccagaagtc

1441 atctcaccaa gaggtgccca aggagtggtc ggtaactggg gtgaagtcgt aacaaggtag

1501 ccgtagggga acctgcggct ggatcacctc ctttctaagg a
